# #Sponseredathlete: the marketing of image and performance enhancing drugs on Facebook and Instagram

**DOI:** 10.1007/s12117-023-09491-4

**Published:** 2023-03-28

**Authors:** Nick Gibbs

**Affiliations:** Northumbria University, Lipman 032, 2 Sandyford Rd, Newcastle upon Tyne, NE1 8SB UK

**Keywords:** Image and performance enhancing drugs (IPEDs), Social media, Online drugs market, Anabolic steroids, Digitisation, Health and fitness industry

## Abstract

This article sets out to investigate the marketing of image and performance enhancing drugs (IPEDs) on the social media platforms Facebook and Instagram. Drawing upon a ‘connective’ ethnographic exploration of IPED use and supply, the paper first outlines a supplier typology on these platforms, before shedding light on the marketing strategies employed by sellers in order to overcome the inherent distrust of online sales and build a trustworthy brand. Techniques identified include athlete sponsorship, the sharing of bodybuilding fitspiration content, self-objectification, posting images showcasing transformation photos and customer feedback, and seasonal sales and promotions. Analysis encompasses the centrality of product branding, the overlaps between licit and illicit market advertising strategies, and the affordances of the platforms under study. Finally, conclusions relating to the implications of these findings to scholarship, policy, and regulation are offered.

## Introduction

The use and supply of image and performance enhancing drugs (IPEDs) has come under sustained scholarly scrutiny over the last decade (Evans-Brown et al. 2012; van de Ven, Mulrooney and McVeigh 2019), with a particular focus on the burgeoning online market for substances employed to bolster athletic performance, physique, and wellbeing (Antonopoulos and Hall 2016; Hall and Antonopoulos 2016; Fink et al. 2019; Gibbs et al. 2022a). Image and performance enhancing drugs, for the remit of this article, are ‘substances that enhance muscle growth and reduce body fat’ (Underwood 2017: 78). This work has therefore opted to focus on what the Human Enhancement Drugs Network (HEDN) calls ‘Muscle Drugs’ and ‘Weight-loss drugs’ (HEDN 2020). HEDN identify the most common muscle drugs as anabolic androgenic steroids (AAS) and human growth hormone (hGH) as well as highlighting the polypharmacy undertaken by most muscle drug users with, amongst other types of enhancers, weight-loss drugs like clenbuterol and human chorionic gonadotropin (HCG) consumed alongside the muscle-drugs themselves.

Anabolic androgenic steroids are a class of drugs that include the male hormone testosterone, or a synthetic derivative of it, that are commonly employed to bolster muscular performance and strength, improve athletic ability, and achieve a lean muscular appearance (Christiansen 2020). These substances, which are the most commonly used IPEDs (Begley et al. 2017), are ordinarily consumed as part of a ‘cycle’, whereby a course is taken in a set time period (typically eight to twelve weeks) before the user is ‘off-cycle’, wherein they assume a period of abstinence (Evans-Brown et al. 2012) and post-cycle therapy (PCT). PCT describes taking drugs and supplements to mitigate or reverse negative side effects of AAS and restore natural hormone levels in the body (Christiansen et al. 2017).

Turning to the IPED market, although scholarship has long noted its peer-reliant, culturally embedded nature (Coomber et al. 2015; Coomber and Salinas 2019), more contemporary research has astutely outlined online supply over hardcore fitness forums (Turnock 2021a), online pharmacies (OPs) (Cordaro et al. 2011; Corazza et al. 2014; Mackey and Nayyer 2016; van de Ven and Koenraadt 2017), and social media sites (Gibbs, Forthcoming) as the landscape has notably shifted from offline, gym-based sales to a hybrid online-offline model (see Turnock 2021a).

The digitisation of IPED supply falls within a broader change in consumption from hardcore ‘expert’ type users (Christiansen 2020) to a less culturally embedded user base who, in the absence of peer networks, rely on ‘decentralised […] open to anyone’ (Antonopoulos and Hall 2016: 708) online spaces to easily access a range of potentially harmful substances, often sold by highly profit-driven market-oriented dealers (Fincoeur et al. 2015). Importantly, this is not to say that traditional offline markets have ceased to exist. Instead, the UK IPED marketplace can be conceptualised as a dual space, wherein hardcore fitness networks provide culturally embedded users with readymade supply chains (Coomber et al. 2014; Antonopoulos and Hall 2016; Coomber and Salinas 2019), whilst online spaces afford access to those lacking these community connections (Gibbs et al. 2022a). Importantly however, this is not to say that overlap does not exist between these two aspects of the market and, as will be discussed in this paper, the online often bleeds into the offline as well as offline norms and cultures manifesting in online spaces (Gibbs and Hall 2021).

However, whilst the function and user demographic of ‘new and novel’ (Salinas et al. 2019: 50) online markets has been somewhat addressed in the literature, scant research has examined the marketing strategies that are employed by digital suppliers (for exceptions, see Mackey and Nayyar 2016; van de Ven and Koenraadt 2017). In response to this lacuna in the current scholarship, this article draws upon a ‘connective’ ethnographic (Gibbs and Hall 2021) exploration of image and performance enhancing drug sellers on the social media sites Facebook and Instagram to first outline supplier types on these platforms, before shedding light on the marketing strategies employed by these actors. Analysis will encompass the centrality of product branding, the inherent distrust of social media sales, the importance of building a trustworthy brand online, the overlaps between licit and illicit market advertising strategies, and the affordances of the platforms under study in order to enhance knowledge of this rapidly evolving online marketplace.

### The IPED market

The market for image and performance enhancing drugs has undergone seismic changes in the last two decades and, as has been alluded to above, its partial digitisation has created something of a dual space of commerce (Gibbs, Forthcoming). Before presenting an overview of the supply-side of the market however, it is worth considering the production of IPEDs. According to Turnock (2020), most anabolics steroids in the UK are produced in domestic ‘underground labs’ (UGLs) (Coomber et al. 2015), with raw powders imported from ‘producer’ countries like China, Egypt, Greece, Thailand, Turkey, and India (Antonopoulos and Hall 2016; Brennan et al. 2018; Llewellyn and Tober 2010; Denham 2019). Underground labs can be defined as illicit operations where IPEDs, most commonly anabolics androgenic steroids, are produced from these imported powders outside of registered pharmaceutical manufacture. These operations vary in scale from minor set-ups with minimal expertise, to large-scale production staffed by highly specialised (but illicitly operating) staff capable of producing a wide range of enhancement drugs beyond just AAS (see Gibbs Forthcoming, for an overview of the UK UGL market). The public health challenges of such production are widely reported, encompassing product dosing, hygiene, and contamination (Llewellyn and Tober 2010; Coomber et al. 2015). Analogous substances like post-cycle therapy drugs and human growth hormone, on the other hand, are generally produced in licit pharmaceutical laboratories in jurisdictions with more lax regulations and subsequently imported or removed from domestic pharmaceutical supply chains by illicit actors (Fink et al. 2019).

As has been alluded to above, empirical evidence indicates that the bulk of IPED transactions occur between peers operating within sporting or fitness-related contexts (van de Ven and Mulrooney 2017; Sagoe et al. 2014), and can broadly be characterised as social supply (Coomber et al. 2014; Begley et al. 2017). Accordingly, literature around IPED supply tends to ascribe a pivotal role to one’s social network or community (Bates et al. 2017; Greenway and Price 2018). Coomber et al. (2014) note that their participants overwhelmingly sourced IPEDs from ‘connected friends’, whilst Fincoeur et al. (2015: 241) suggest that some suppliers are subject to a process of normalisation due to their IPED-using peers, and thus do not view themselves as ‘real dealers’. In this sense, they conceptualise the suppliers in their sample as ‘cultural products’ who engage in a process of ‘cultural reciprocity’ in consumer-supplier interactions, whereby financial gain is less important than other benefits (e.g., respect, reputation, or acknowledgement of expertise) within an ‘enduring relationship’ between the two parties (Fincoeur et al. 2015: 242; van de Ven and Mulrooney 2017). In practice, Antonopoulos and Hall (2016: 707) note that social supply tends to occur in ‘discreet places’ in gyms, such as toilets and changing rooms, and sellers rarely approach prospective customers, relying instead on their personal network of trusted users. However, Coomber and Moyle (2014) critique the concept of social supply on account of its dismissal of the fundamentally economic nature of drug transactions. Addressing this, they coined the term ‘minimally commercial supply’ to acknowledge the inevitable economic exchange taking place.

Crucially, access to this ‘partial’ offline market (Fincoeur et al. 2015) is guarded by symbolic barriers, meaning that those without the prerequisite cultural or bodily capital cannot gain access (Maycock and Howat 2005; Coomber and Turnbull 2007; Antonopoulos and Hall 2016). However, whereas these cultural battlements have traditionally limited the consumer base to community embedded ‘expert’ users (Christiansen 2020), discernible shifts have occurred that have ushered in a new wave of prospective buyers. Firstly, a raft of literature points to a professionalisation of the IPED market. Fincoeur et al. (2015), drawing upon the axes of commercialisation and cultural embeddedness, contend that IPED supply has become more professionalised and profit driven. This is supported by Salinas et al. (2019), who found that their sample’s consumption ‘transcended IPEDs to encompass a much broader cocktail of substances’ (Salinas et al. 2019: 49) including recreational drugs like cannabis and cocaine (see also Turnock 2021b). As such, this polydrug use is facilitated by increasingly ‘market-oriented dealers’ (Fincoeur et al. 2015: 244), with fewer ties to the fitness community and, crucially, with no qualms about selling to non-culturally embedded customers. Secondly, changes in the global accessibility of IPEDs have occurred due to the rise of online selling. Hall and Antonopoulos (2016: 64) note that the market ‘has in some respects moved online’, and therefore customers who lack cultural and bodily capital can circumnavigate traditional barriers to supply. Such customers, termed ‘occasional users’ (Antonopoulos and Hall 2016: 702), are the principal target for online sellers, who can offer a ‘virtual relationship’, mimicking that of the gym community. As a result, the authors describe the online IPED market as ‘decentralized, highly flexible with no hierarchies, and open to anyone’ (Antonopoulos and Hall 2016: 708). Finally, recent scholarship has examined changes in the motivation to use IPEDs and the impact of a focus on medicalised ‘wellbeing’ rather than conventional sporting consumption (Underwood et al. 2021). This includes users sourcing products online as self-prescribed testosterone replacement therapy (TRT) (Dunn et al. 2021; Harvey et al. 2021; Turnock 2022) as part of a wider pharmaceuticalisation of health (Williams et al. 2011; Nettleton 2013; Morrison 2015).

As was set out in this work’s introduction, this online space has sprawled over surface web forums, online pharmacies, and social media. Scholarly research into social media drug supply, although still relatively scant, has grown substantially in recent years (see Moyle et al. 2019; van de Sanden et al. 2021; Moeller et al. 2020; Bakken 2021; Oksanen et al. 2021). Examining the social media market for cannabis, cocaine, and prescription medications, Demant et al. (2019) found that sellers advertised on open platforms like Facebook, before negotiating deals via encrypted apps like Wikr. Further, the researchers identified that the Facebook groups through which sellers advertised their products tended to be open to the public and therefore sellers were, on the whole, overt about their enterprise. In relation to IPEDs, Mackey and Nayyar (2016) highlight the use of social media marketing on platforms like Facebook, YouTube, and Twitter by rogue OPs as a means of mirroring consumer patterns. Further, Shukman (2020) notes that, on Instagram, sellers employ hashtags including #anabolic and #performanceenhancement to increase the searchability of their marketing posts, therefore allowing consumers to simply search for their desired product in much the same way as conventional e-commerce sites. This ease of access reflects Hall and Antonopoulos’ (2016) contention that barriers of entry into the IPED market have been lowered, as users simply require an account and some basic ICT skills to acquire a host of potent substances. It is this context that foregrounds this paper and, in the absence of any substantial prior research explicitly addressing the marketing of IPEDs on Facebook and Instagram (for a recent exception, see Cox et al. (2023), it is hoped that fresh light can be cast upon the solicitation of prospective online customers on these platforms.

### The digitised health and fitness industry

Alongside an understanding of the contemporary IPED market, it is also crucial to explore the licit health and fitness industry and its digitised advertising apparatus in order to contextualise the IPED marketing strategies that will be set out below. As has been well-documented, the health and fitness industry has experienced a meteoric growth in the last two decades (Smith Maguire 2008; Sassatelli 2010; Millington 2016; Christiansen 2020) as evermore primacy is placed on the physical form as a site of identity formation and consumption in the leisure economy (Kotzé and Antonopoulos 2019; Gibbs et al., 2022b). Alongside gymnasia, this market houses a range of goods and services including health supplements, wearable fitness monitoring devices, personal training, diet planning, CrossFit, and activewear (Cederström and Spicer 2015; Crockett and Butryn 2018; Andreasson and Johansson 2019). Speaking to the breadth of this ‘dumbbell economy’ (Ellison 2018), the market is worth £5 billion in the UK alone and, despite being heavily disrupted by the COVID-19 pandemic (Gibbs 2021; Du et al. 2020), remains a central pillar of the leisure economy (having remained in ‘remarkably good shape’ with just a 2.4% reduction in value compared to pre-pandemic figures (Marcellin 2022)).

More presciently, the various lockdowns and limitations on physical space accelerated the already burgeoning online fitness market (Nyenhuis et al. 2020; Godefroy 2020; Cantalani et al. 2021; Zhu et al. 2022) and led to something of a digitisation of health and fitness, particularly on social media (Jong et al. 2016). Social media and health and fitness coalesce in the form of ‘fitspiration’, which includes ‘fitness-related images and/or text intended to inspire people to pursue a lifestyle of fitness and health’ (Fatt et al. 2019: 1313). This typically involves users documenting their workouts alongside motivational quotations and training advice. Such content, as well as being posted by digital ‘prosumers’ (Yar 2012; Hall 2019), is frequently disseminated by fitness influencers and online coaches or personal trainers (Toffoletti and Thorpe 2021), often leading to negative self-image in social media users (Fardouly and Vartanian 2016; Tiggemann and Zaccardo 2018; Tiggemann and Anderberg 2020). Further marketing tactics noted within the digital health and fitness market include personal trainers and online coaches posting ‘before and after’ or ‘transformation’ images of their clients, in order to promote the effectiveness of their services (Parasecoli 2005; Basabain et al. 2021), digital influencers establishing a relationship with a health and fitness-related company as a brand ambassador or sponsored athlete (Silva et al. 2021), and the utilisation of in-app commerce (particularly on Instagram) to simultaneously advertise and sell products and services (Instagram 2022).

Given the above outline of the partial digitisation of the IPED market, this article sets out to explore the marketing strategies employed by IPED sellers on social media platforms, and any potential synergies or replication between the licit and illicit economy. Indeed, MacKenzie (2020: 2) propounds that ‘[i]llegal business dances to very much the same tune as legal business, using similar methods, having similar aims, and achieving similar ends’. Therefore, given that the same fundamental injunction to turn a profit binds the licit and illicit economy, it stands to reason that social media IPED suppliers share some of these methods in their selling efforts.

## Methodology

This paper draws on data collected as part of a wider ‘connective’ ethnographic study (Leander and McKim 2003; Gibbs and Hall 2021) examining the use and supply of IPEDs in both online and offline contexts. The year-long mixed methods study – conducted between 2019 and 2020—encompassed online and offline ethnographic observation, which saw the author train alongside participants around five times a week, conduct twenty-eight semi-structured interviews with gym users and those involved in the consumption and supply of IPEDs, undertake digital ethnography on Facebook and Instagram, and conduct online interviews with IPED suppliers. Given that the project coincided with the COVID-19 pandemic and the subsequent national lockdown(s), follow-up interviews with the most committed participants (*n* = 5) were also carried out using video conferencing software in November 2020 (see Gibbs 2021).

This approach echoed the inherently ‘messy’ nature of criminological ethnography (Liebling 2001; Treadwell 2020) and, it is hoped, that the connective ethos – wherein the traditional online/offline dualism was disregarded (Prince 2019) – brought the researcher far closer to a state of verstehen (Ferrell 1997) than other, more one-dimensional means of data collection. Data variously took the form of interview transcripts, 160 pages of digital ethnographic screenshots, and offline fieldnotes. These were then thematically analysed. Participants were initially recruited using purposive sampling on social media, followed by a snowball approach. Pseudonyms were assigned to anonymise their identities, and the names of IPED brands as well as any identifiable information in the dataset were redacted (as is demonstrated in the various pictorial data presented below). Ethical approval for the research was granted by Northumbria University in 2019 and a process of ‘contextual integrity’ (Nissenbaum 2010) – or situational ethics – was employed to assess whether data sat in the public realm and, where possible, informed consent was gathered through the use of formal consent forms. Given that the field of digital research ethics is still in its relative infancy (Abidin and de Seta 2020), such projects cannot rely on a ‘one size fits all’ approach (Gatson 2011) and therefore, following Beninger et al. (2014) and Alim (2014), data content posted on open, publicly accessible platforms were considered to be in the public realm. Finally, unlike Demant et al. (2019) the social media profiles created explicitly stated the researcher’s identity, institutional affiliation, and image so as to avoid any deceptive practices.

## Findings

### Seller typologies

The project’s exploration into IPED supply on Facebook and Instagram identified two ‘types’ of sellers: ‘UGL representatives’ and ‘independent resellers’. Although this unsubtle typology does not necessarily capture the heterogeneity of actors involved in the social media supply chain, it helps to provide a glimpse of the landscape of the online market and therefore frames this article’s analysis (for a similar typology of IPED sellers on surface web forums, see Turnock 2021c).

First, underground laboratory representatives – who supply most prevalently on Facebook—sell exclusively on behalf of the UGL they are affiliated with, acting to promote a specific brand. In reward, they are offered commission for their sales. Certain brands opt to be more visible than others on social media and, over the course of the fieldwork, the underground lab representatives most frequently encountered were mainly from Phoenix Labs, Energise, Victory Labs, and KSI. Their modus operandi generally involves creating private Facebook groups through which to advertise their products and offer advice and guidance (see Gibbs, Forthcoming). In-keeping with the UGL market, these sellers tend to only sell AAS rather than other medicinal products. Further, unlike the actors that Hall and Antonopoulos (2016) identified in the illicit medicines trade, UGL representatives tend to be British as a result of the domestic nature of the underground lab economy and, unlike most recreational drug sellers on social media, they are open about their identity within the groups that they operate.

Unlike UGL representatives, independent IPED resellers may carry various different UGL brands and different medicinal products and therefore do not necessarily represent a specific producer. With that said, most of these suppliers appear to have long-standing relationships with certain online pharmacies or underground labs and (although not exclusively) stock and advertise their products. Independent resellers appear more drawn to Instagram as a platform rather than the largely UGL-operated Facebook groups and, in distinction to the underground lab representatives’ apparent disregard for their anonymity, they typically conceal their identity with generic profile pictures of bodybuilders or pharmaceutical imagery and employ IPED-related pseudonyms (see Demant et al. 2020; Bakken, 2021). For this reason, their nationality is unclear from their profiles. However, following numerous interactions with such sellers, data collected in this project suggests that most operate from nations like China, Pakistan, Turkey, and Ukraine (see Hall and Antonopoulos 2016) (Table 1).

**Table 1:** Captures the main characteristics of these seller types

Seller type	Preferred platform	Anonymity	Products sold	Geographic location
Underground lab representative	Facebook (specifically the Groups feature)	Typically use authentic images and names	One specific UGL brand	Britain
Independent reseller	Instagram	Typically anonymise images and names	Range of UGL brands and (counterfeit or genuine) pharmaceutical-grade products	Large variance, typically Southeast Asia and Eastern Europe

Given the digital architecture of Facebook and Instagram, both seller types interact with customers using each platform’s built-in messaging service, providing a product ‘price list’ before engaging in an ‘online haggle’ (Terwiesch et al. 2005) in an example of ‘peer-to-peer’ negotiation (Turnock 2021a). Interaction is then typically transferred to an encrypted messaging service like WhatsApp, Kik, or Wikr (see Moyle et al. 2019), where payment and shipping are discussed (for a full account of this, see Gibbs, Forthcoming).

Important to note however is that this seller typology is certainly not comprehensive, and some actors might move between these categorisations or indeed overlap the two. With this in mind, echoing Christiansen et al.’s. (2017) typology of AAS users, this article has employed the Weberian notion of an ‘ideal typology’ to capture the core characteristics of each seller type, rather than necessarily describing ‘real-world’ individuals (Hekman 1983). Therefore, the two categories of social media seller ought to be viewed as an approximation of two market positions observed within the broader research project, which may be refined following further study.

## The importance of brand identity and the need to appear legitimate

An overarching theme of this research paper is the motivation of the suppliers’ marketing tactics, which cohere around two themes: to build a recognisable brand, and to present themselves as trustworthy to prospective consumers. Speaking in relation to the UK underground lab market, IPED harm reduction specialist and ‘anabolics coach’ (Gibbs et al. 2022a) Rob emphasised the need to ‘*build a reputation*’ given users’ proclivity to ‘*go to a lab because it’s got a good reputation and it produces good quality drugs*’, therefore, ‘*UGLs, unlike any other drug, and the closest similarity would be ecstasy, rely on brand recognition*’. Echoing the often eye-catching branded presentation of ecstasy and other drugs like LSD then (Duterte et al. 2009), how an IPED product is packaged and advertised is key to understanding customer choice. This sentiment can be extended from UGLs to independent IPED resellers who, given the inherently untrustworthiness of illicit drugs markets (Tzanetakis et al. 2016; Bancroft et al. 2020), must also attempt to level out the power differential – or information asymmetry (Beckert and Wehinger 2012)—between buyers and sellers of illicit goods online by presenting themselves as genuine and authentic (Décary-Hétu and Leppänen 2013; Holt et al. 2016; Moeller 2018; Koenraadt 2019).

The notion of branding and self-presentation by illicit drug dealers on social media has recently been addressed by Bakken (2021), who analysed illicit drug supplier Facebook profiles in Scandinavia to shed light on how individuals who sold cannabis, ecstasy, and other controlled substances, use their profiles to enhance their business. Leaning on Gambetta’s (2009) interpretation of signalling theory, Bakken differentiates between ‘signals’ – observable characteristics displayed to build trust – and ‘signs’ – non-curated features of a profile that might bolster kinship with the buyer – to establish a typology of ‘professional’, ‘personal’, and cultural’ presentation types, which ultimately attempt to entice prospective customers and build cultural proximity and trust (see also Demant et al. 2020 for a discussion of illicit medicine sellers’ profiles on social media). Similarly, Hämäläinen (2019), exploring drug vendor usernames on darknet market AlphaBay, found that the linguistic choices made by sellers are crucial to their online persona and outward perception. Their research uncovered a suite of differing approaches to username brand creation from illicit drug sellers, including sleek professional presentations, plagiarising real-life brands and characters, and explicit controlled drug references.

Besides the online presentation of drug seller profiles on social media, promotional strategies like providing ‘freebies’ (Coomber 2003; Ladegaard 2018), quality testing (Bardwell et al. 2019; Turnock 2021a), and showcasing customer reviews (Van Hout and Bingham 2014; Broséus et al. 2016) have also been identified in illicit online drugs markets. Notably, many examples of this research have focussed on dark web spaces and sellers concerned with ‘traditional’ recreational drugs like ecstasy and cocaine (see for example Nurmi et al. 2017). Therefore, this article aims to track the adaptations and crossovers with these tactics found on the Facebook and Instagram IPED markets in order to add to the existing literature. With that said, precedent has been somewhat set for this work by van de Ven and Koenraadt’s (2017) study of buyer–seller relationships on surface web online pharmacies. Though not examining social media supply, the authors found evidence of ‘quality websites’ which employed ‘responsible vending’ strategies (Van Hout and Bingham 2014) like providing product information and guidance (sometimes delivered over the telephone), extensive research and testing, and sourcing from reputable wholesalers. van de Ven and Koenraadt (2017: 53) conclude that these ‘customer service’ measures are vital in building and sustaining trust (and therefore business) and echo traditional offline ‘social supply’ models (van de Ven and Mulrooney 2017; Begley et al. 2017) despite their digitised means. However, although van de Ven and Koenraadt (2017) provide excellent insight into customer-seller trust building on OPs, sellers’ more explicit marketing and advertising strategies are overlooked. Therefore, this article aims to flesh out the authors’ initial exploration and focus on the means by which IPED sellers simultaneously market their products and build consumer trust.

A further consideration, however, is the illicit nature of the products on offer and therefore the underlying awareness of law enforcement. This is documented by Lusthaus (2012) in his exploration of trust in cybercrime networks. Unlike the licit economy, where digital marketing teams unproblematically seek maximal exposure (Pauwels and Dans 2001), illicit online actors must not be overly explicit in their customer soliciting, especially on surface web platforms (Bancroft et al. 2020; Bakken 2021). This is termed the ‘transparency paradox’ by Tzanetakis et al. (2016), who critically examine the balance between exposure and visibility (see also Demant et al. 2020). This is addressed by Bakken and Harder (2022) in their comparison of licit and illicit cannabis markets on social media. They found that illicit actors, in countries where cannabis is not legalised, opted not to use images or showed only blurry, functional content rather than any market gloss, and were business-like and impersonal in nature. However, the wider IPED market is generally poorly regulated and is certainly not a high priority for law enforcement (Gibbs et al. 2022b). As such, sellers might exhibit riskier marketing behaviours compared to their more heavily regulated counterparts in recreational drug supply. Finally, Bakken (2021) notes the lack of a formalised feedback system on social media sites compared with dark web and surface web forum illicit drug supply (Hardy and Norgaard 2016; Turnock 2021a), which is particularly challenging given that online markets require democratised customer review systems in place of relationships and social capital (Tzanetakis et al. 2016). As such, the strategies to maximise customer rapport and establish credibility presented below are of paramount importance to this market.

What follows will first address the marketing tactics exhibited by underground laboratory representatives on Facebook and Instagram, before attention is turned to independent resellers’ strategies for building a reputable and trustworthy brand. This will be followed by a discussion focussing on the overlaps between the licit and illicit ergogenic aids industries, the affordances of the social media platforms under study, and the implications for those regulating the IPED market.

## Underground laboratory marketing strategies

### Athlete sponsorship

The first means by which underground labs build brand recognition and establish trust is the use of sponsored athletes. Hall and Antonopoulos (2016: 39), writing in relation to the sale of fake medicines online, note that ‘[v]irtual ‘word of mouth’ can play an important role in terms of establishing, assuring, and circulating the legitimacy of a seller and quality of the service on offer’. As such, UGL representatives are aware of the sanctity of creating promotional networks that can vouch for their trustworthiness. The most potent means of achieving this is to employ ‘independent’ sponsored athletes to market on behalf of the lab on social media. This arrangement echoes the licit phenomena of the social media influencer (Marwick 2015; Lawrence 2022) wherein prominent users are selected as brand ambassadors, promoting certain brands in exchange for ‘freebies’ (Turnock 2020). It should be noted here that sponsored athletes are distinct from UGL representatives as, whilst both of these actors operate on behalf of their underground labs, brand ambassadors are not involved in the actual sale of illicit products and instead function to offer advice, guidance, and promotion. Therefore, underground labs select influential figures in the fitness community and renumerate them for showing public affiliation to the lab. This was explained by Sam in relation to his recent appointment as a Phoenix Labs ‘brand ambassador’:

‘S: A *lot of the labs have sponsored athletes now* […] *the guy who owns the lab basically got in contact with me saying, ‘I know you’ve got a large client base, do you want to be a sponsored athlete?’. Then obviously in turn my clients use the brand.*

*N: Do you get any sort of financial discount for doing that?*

*S: Yeah, he’ll do me my cycles essentially for no cost. It is a good deal, but then obviously I’ve got to get them customers. It’s not a certain amount of clients or anything but I’ll need to refer any of my clients who are on cycle or going into a prep*^1^* to him. That’s basically the deal, which works pretty well.*’

As Sam demonstrates, UGL Phoenix Labs replicate well-known fitness brands like Gymshark, gifting free products to users with a substantial ‘*client base*’. The UGL are clearly aware of their target market and therefore judge Sam’s personal consumption to be less costly to finance than the potential benefits of his extensive coaching clientele. Beyond this however, the paramount importance of brand identity is reflected in Phoenix’ strategy, as they strive to foster a symbolic attachment with Sam’s clients, whilst also utilising his reputation as a coach to promote their own legitimacy and sporting affiliation.

Although this behaviour was observed in other UGLs, Phoenix dominated the social media market with their sponsorship efforts, as amateur bodybuilder and sponsored athlete Carl explained, ‘[s]*o if you have a big name in the industry and people know you,* [Phoenix] *will sponsor you. That is across the board, I mean they must sponsor twenty or thirty athletes that I know in one Facebook group*’. This claim was supported by Rob, who asserted that ‘*they sponsor about fifty athletes*’ in total, ranging from IFBB Pro bodybuilders^2^ to amateur gym users with a sizable social media following. The latter of these categories is epitomised by Carl, an amateur forty-six-year-old bodybuilder from Wales, who held a number of UGL sponsorships, including Phoenix, during the data collection period. Despite Phoenix’ reputation as a ‘*very elitist group*’ in Carl’s eyes, he explained his worth to the UGL:

*‘So the guy sitting on the sofa weighing eighteen stone who needs to get in shape, I’m more appealing to him because that’s what I did. The huge bodybuilders who compete every year, their physiques, although everyone fantasises about having them, they’re reasonably unattainable. So to give people some perspective and some actual ‘I went from this to this in nine months using this product and this training programme and this diet’, people just want those items that you’ve been using*.’

Though Phoenix Labs are primarily concerned with elite competitors, Carl’s sponsorship functioned to mainstream the brand. This speaks to the democratisation of the IPED market (Fincoeur et al. 2015; Hall and Antonopoulos 2016), as the ‘*guy sitting on the sofa weighing eighteen stone*’, who presumably lacks personal connections in the industry, is able to use Carl as a proxy and, in turn, is more inclined to purchase IPEDs in order to make a similar transformation. In this sense, Phoenix benefit from Carl’s ‘signs’ of authenticity (Gambetta 2009) as a culturally proximate figure and therefore someone who can be trusted. Digging deeper, writing in relation to the licit economy, Abidin (2017) terms this appeal ‘calibrated amateurism’, which showcases a deliberately unpolished relatable persona rather than a traditional commercial skin (De Veirman et al. 2017; van Driel and Dumitrica 2021). Carl’s amateur fitness journey, which was meticulously tracked through his Instagram account, therefore represents his brand (Khamis et al. 2017) which he mobilises in order to receive ‘*five percent off the price list’*. Further, referencing several famed bodybuilders that were continually linked with Phoenix throughout the fieldwork, Carl noted that ‘[t]*he problem with those guys is* [that] *they come and go very quickly and of course when they leave, they leave with massive amounts of people*’. Lesser-known brand ambassadors like Carl thus represent a safe bet for UGLs in the increasingly competitive online IPED market.

Most brand ambassadors appear to be approached by their sponsors. This is particularly true of participants with a high degree of influence in the IPED community like Rob, who incredulously recounted his experiences of being solicited by Phoenix:

*‘I’ve been approached numerous times by labs saying, ‘if you say we’re good we’ll give you loads of free gifts’, but I’m not interested. If I know a lab’s good then I’ll say it’s good. Phoenix sent me fifty vials of their gear for no apparent reason whatsoever. I phoned them up like, ‘what have you sent me this fucking shit for? I won’t use it’. They said, ‘nah we just wanted to show you how good it is’.* […]* They’ve done that a lot and it’s odd, it’s just odd behaviour.’*

Although Rob turned down Phoenix’ offer, the UGL’s unsolicited delivery illustrates the importance placed on brand ambassador marketing by the lab and their astonishingly overt approach. The significant expense undertaken for such a gesture speaks to the value of onboarding well-respected industry professionals like Rob, whose endorsement would dramatically bolster Phoenix’ brand integrity and signal their legitimacy.

**Fig. 1:**
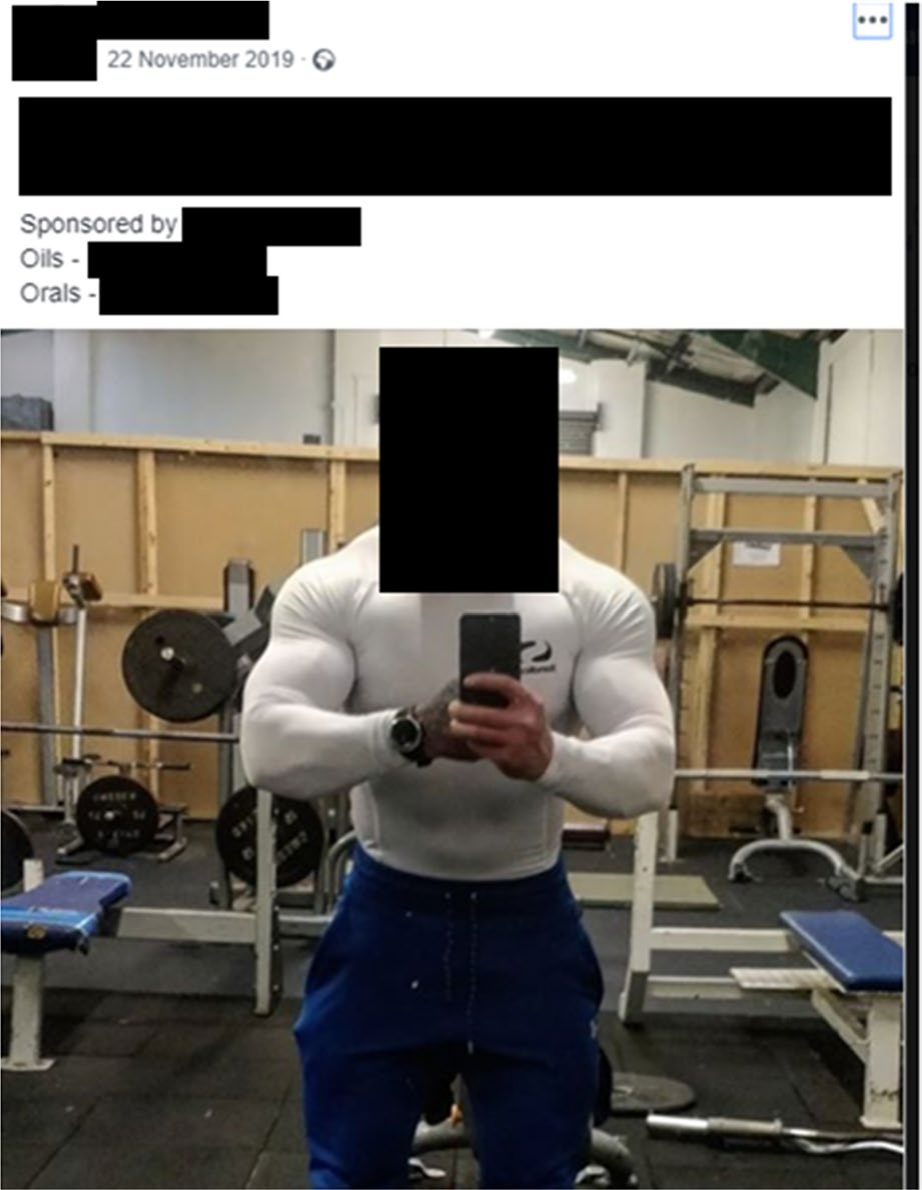
An amateur Facebook bodybuilder publicly showing his affiliation with UGL Energise (22/11/19)

**Fig. 2:**
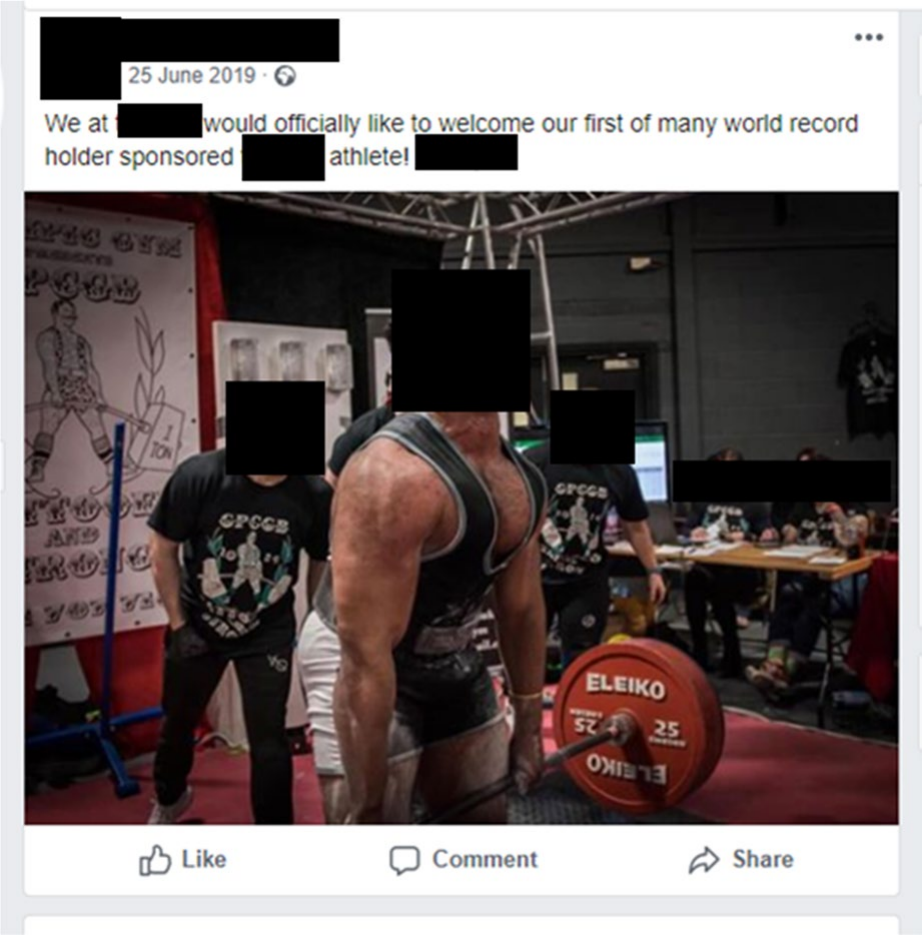
Victory Labs celebrating the achievements of one of their sponsored athletes via their official Facebook page (25/06/19)

Sponsored athletes perform a number of roles depending on the UGL’s brazenness and their own willingness to engage in the IPED market. The most overt of these methods are exemplified by Carl, who stated that, ‘*my form of promotion is hashtagging in posts, bigging up the lab, speaking to people who message me privately who ask me what I take. And I then push them towards the lab that way*’. This is reminiscent of licit social media influencers, for whom maintaining followers’ attention and engagement are of paramount importance to their financial interests (Hearn 2010; Marwick and Boyd 2011; Baker and Walsh 2018). For Carl, the UGL brand name is included in his Instagram and Facebook content, typically in the format #teamphoenix. This highly public advertising, operationalised by various UGLs (see Figs. 1 and 2), illustrates the lack of regulatory oversight on social media, as Sam mused that Phoenix are ‘*playing a very fine line with getting caught, but at the same time do law enforcement really care about it?*’. Whilst the inclusion of brand names in public posts may appear reckless, unlike general drug hashtags like #buysteroidsUK, underground lab brand names require a degree of cultural embeddedness to access. For this reason, as long as IPED regulation remains ‘*way down the pecking order*’ (Luke), law enforcement agencies lack the community-specific knowledge necessary to successfully locate the posts.

Publicly available hashtagging appears to be more prevalent in amateur brand ambassadors, compared to IFBB Pros or other elite athletes. This is perhaps due to sponsored athletes like Carl’s relatively modest platform (under a thousand followers on Instagram) compared to Sam’s more than three thousand followers, coupled with his repute in the licit fitness industry. This was addressed by amateur bodybuilder Jake during a follow up interview in late 2020, where he was adamant that, ‘*you would never be asked to put it on social media. Like I would never put on Instagram like, ‘go and buy this’*’. Clearly, Jake’s burgeoning reputation as a bodybuilder, alongside his employment as a security guard and model would, in his mind, preclude him from undertaking the public marketing demonstrated by Carl and, just like Sam, overt sponsorship would represent a far greater professional risk for him.

Despite his unexceptional following however, Carl compensated for his limited reach with his trust-building potential. On this, he contended that ‘*it’s all done by trust really, which is where we come in, us sponsored athletes’.* Emphasising the parallels between the licit fitness industry and the illicit IPED market, Bakken and Harder (2022: 4) suggest that the ‘entrepreneurship of influencers relies on forming affective relationships with followers based on conceived proximity’, and therefore building trust – or ‘digital intimacies’ (Reade 2021: 2)—on the premise of biographic convergence or shared experience is vitally important (Abidin 2015; Duffy 2017). Echoing licit fitness influencers then, Carl functions as a guarantor of quality and legitimacy as his bodily progression is testament to his successful IPED use, facilitated by the UGL. Therefore, by acting as the public face of the lab, brand ambassadors afford the operation a heightened level of cultural embeddedness, attempting to replicate the offline models of social supply (van de Ven and Koenraadt 2017; van de Ven and Mulrooney 2017) in that sales are based on tailored, holistic consultation rather than faceless online interaction (see Gibbs et al. 2022a). Whilst such tactics can be interpreted as a mere profit maximisation exercise, this is perhaps a reductive reading. Having since moved away from Phoenix Labs, Carl represented Inception, a smaller British UGL, at the close of the data collection period,^3^ and was keen to portray their genuine, well-intentioned marketing approach:

‘*Their attitude and their business plan is far more my way of thinking. Less pressure, if you want to buy you can buy but a lot of advice is offered, free advice. They asked for their sponsored athletes to offer free advice on the* [closed Facebook] *group, you know, not to charge for things because they’re paying you to do it. They’re far more my way.’*

UGL Inception demonstrate a responsible vending approach (Van Hout and Bingham 2014), encouraging their sponsored athletes to offer free advice around nutrition, training and safe IPED use. Alongside overt brand affiliation signifiers like hashtagging, UGLs also utilise brand ambassadors in a more subtle, community-embedded manner. This is portrayed in Sam’s experience of working with Phoenix:

‘*N: Does* [your sponsorship] *involve you posting promo stuff or wearing branded gear or anything?*

*S: It’s not about wearing anything or stuff like that, which is what I find quite strange. It’s more like people in the gym will ask you, and then you’ll point them in the direction of Phoenix. It is quite a common thing, like if Phil Heath* [a well-known American bodybuilder] *was training in your gym you’d want to know what brand he uses, because then you know that one works. So yeah, that’s what he* [Phoenix’ owner] *goes off, people openly talking in gyms about it, because they do.*’

As demonstrated, although social media was used to recruit Sam initially, the bulk of his referrals occurred offline in the gym setting. This view was reflected by Rob, as he contended that ‘*sponsorship has always gone on, but it’s definitely more blatant now with social media*’. Thus, capitalising both upon the gradual normalisation of IPED use in the gym and the burgeoning social media market (Richardson et al. 2019; Turnock 2021a; Gibbs Forthcoming), Phoenix effectively have a footing in both spaces, in many ways reflecting the contemporary IPED market. However, whilst UGLs’ employment of brand ambassadors appears to offer the athletes a healthy income supplement, a degree of exploitation is also present. This is exemplified by Carl’s experiences with UGL Energise prior to his affiliation with Phoenix and Inception:

‘*What happened was, I found out that Energise had earned a small fortune off of my back. Basically, I came off the sofa and got into shape, logged a lot of it and they approached me and said ‘we’d like to sponsor you if you promote our products’. They said, ‘we’ll send you free stuff every month, whatever you require, up to a limit of course. Then you need to tag us in every post that you have. Anyone who asks you where you get your products from, push them our way’. Anyway, I found out through a friend that they’d earned in excess of ten thousand pounds from me last summer, and they didn’t pass a penny of it on. I received free stuff every month, it probably cost them about twenty months’ worth of gear and they earned all that off me, if not more. They came back to me and said, ‘we’ll sort you out, we’ll help you out, we’ll give you this or that’, but it never appeared*.’

Carl’s experiences demonstrate the unregulated nature of the IPED market as, unlike the licit economy’s marketing oversight, his exploitation could not be reported or investigated. Having no legally binding contract in place, his relationship with the UGL was entirely based upon trust, which is problematic given the power differential at play (Beckert and Wehinger 2012). This same disparity can lead to brand ambassadors being suddenly excommunicated, as Carl explained when discussing his later experience with Phoenix, ‘*I went to put my third or fourth order in and I was told that I’d been cut off. I was no longer sponsored, I was not promoting the company enough in the way they wanted me to*’. Further, Carl complained, ‘[t]*hey basically wanted everybody to be a seller, and I’m not prepared to sell things. I can promote things but I’m not happy to sell them, I don’t want to risk a jail sentence*’. Carl faced an ultimatum to either risk prosecution or be cut adrift, which ultimately led to him working with Inception in a more advisory role. Finally, this commercial underbelly was further demonstrated during the COVID-19 pandemic. During a follow-up interview with Sam, questioning turned to his sponsorship with Phoenix and whether the arrangement was still in place:

‘*N: How’s it all going with Phoenix? You said last time that you’d recently become sponsored by them. Have you made many referrals?*

*S: It’s funny you’ve said that cus* [sic.] *I was telling Jake the other day, it all seems to have changed at Phoenix. Obviously last time I said to you about getting my cycles essentially for free and all that, well now they’ve got really tight with what they’re giving out. So before I got my whole lot, prep and all, but now I’m lucky to even get the basic compounds. Also, before they just wanted to know that you were passing their details on if anyone asked about becoming enhanced, whereas now they’ve put in a new referral system, which is more trouble than it’s worth in my opinion. So to actually get the products I have to refer X amount of clients, which is just pressure for me as I don’t want to start my lads on anything that I don’t think is necessary or safe. The* [Phoenix] *group chat’s gone a bit quiet too if I’m honest and they seem to have changed ownership - it’s just not the same. Where the lad before would ask us how we’re doing and what we need and all that, it’s a foreign man who runs it now and he doesn’t speak much English. So anyway, I have ended up just going through* [a local supplier] *instead.*’

Phoenix appeared to have been negatively affected by the fall in demand that occurred over the nationwide lockdown (see Gibbs 2021; Zoob Carter et al. 2021; Dunn and Piatkowski 2021) and therefore became less generous with their ‘*freebies*’. The disparity between Sam’s experience as a newly sponsored athlete and this period (which was around six months later) was striking, as the liberal delivery of products he initially received was replaced by a more tightly regulated ration, contingent on an arbitrary number of referrals. Acknowledging the context of this change, Sam concluded, ‘*I reckon they’ve really struggled with the gyms being closed and all the* [competitions] *being cancelled, so I’m not really surprised*’. Ultimately, it appears that the pandemic weakened the ‘responsible vending’ practices (Van Hout and Bingham 2014) previously demonstrated by Phoenix and, as their bottom line was compromised, the lavish giveaways were curtailed in line with the fundamental injunction to turn a profit.

### Fake profiles and deception

Underground labs also employ the more insidious tactic of creating fake profiles and constructing fictitious conversations in order to subversively market their operations. This strategy was summed up by Rob, as he laid out a hypothetical marketing plan that he would follow if he were to set up a lab:

‘*So first I open twenty fake profiles on Facebook or Instagram, join all these bodybuilding pages and I ask a question on my genuine profile or another profile, ‘is this gear any good?’. I then put a picture up of it with it looking all fancy, then within that conversation nineteen people that appear to have no association with each other post how they’ve used it and they find it absolutely fucking brilliant. Straight away I’ve got people wanting to buy that lab because they’ve been on a thread where twenty people have now said, ‘yeah I’ve used it, it’s fucking shit hot’. Bear in mind I’m a UGL owner so I have all day to be on Facebook,* [I would]* do that for a couple of months and everyone believes this is a huge lab with a great reputation, producing a great product. No one knows if I am actually producing a good product, but I’m flying.*

*I’ve seen whole fucking conversations and arguments on Facebook between the same fucking person, long convoluted stories about how a mate got ripped off over a bad lab and he’s now using this and it’s so much better. Not even direct marketing, very very subversive, you know? ‘I bought this’, and it might be a lab that we all know to be a bag of shit like KSI or something, but now he’s got a better lab. But it just comes across as a conversation, to someone reading that, they’re thinking ‘well I’ve used KSI and they’re alright but not great, but with this stuff his gains have massively improved’. It’s all marketing to get people to start seeing this lab’s name popping up again and again*.’

UGLs can exploit the relative anonymity of social media platforms (Schlesinger et al. 2017), taking on multiple identities to weave intricate narratives of the legitimacy and quality of their lab. Such deceptive practices echo the challenges of fake customer reviews on licit e-commerce sites like Amazon and the concerted research and resources that have been mobilised to detect and regulate them (see Paul and Nikolaev 2021; Salminen et al. 2022). This exposes the hypocrisy at the heart of many underground labs’ marketing strategies, as this deception is utilised as a means of encouraging trust in prospective customers, who are, ironically, untrusting due to previous accounts of such trickery. This approach ties into Gambetta’s (2009) notion of signalling, as opposed to the true ‘signs’ of authenticity that exude from the sponsored athletes described above. Here, we can see that attempts at building trust and kinship are entirely curated (Bakken 2021). Hall and Antonopoulos (2016: 40) note that ‘customers tend to be more interested in a product if it is perceived as ‘authentically’ endorsed, rather than a product purposefully promoted by marketer-generated sources’. Therefore, this underhand marketing technique provides what appears to be organic electronic word of mouth marketing (eWOM) (Phua and Ahn 2016) to unknowing social media users. The presence of fake profiles and interactions casts doubt upon the true size of many popular UGLs, as Rob noted that Phoenix Labs’ ‘*social media and online presence would make them appear to be absolutely enormous*’, yet given his above comments, this conclusion may be premature. Ultimately, the use of deception illustrates just how muddy the waters of the social media IPED market are, and the need for vigilance from the consuming population.

## Independent resellers’ marketing strategies

### Bodybuilding fitspiration

**Fig. 3:**
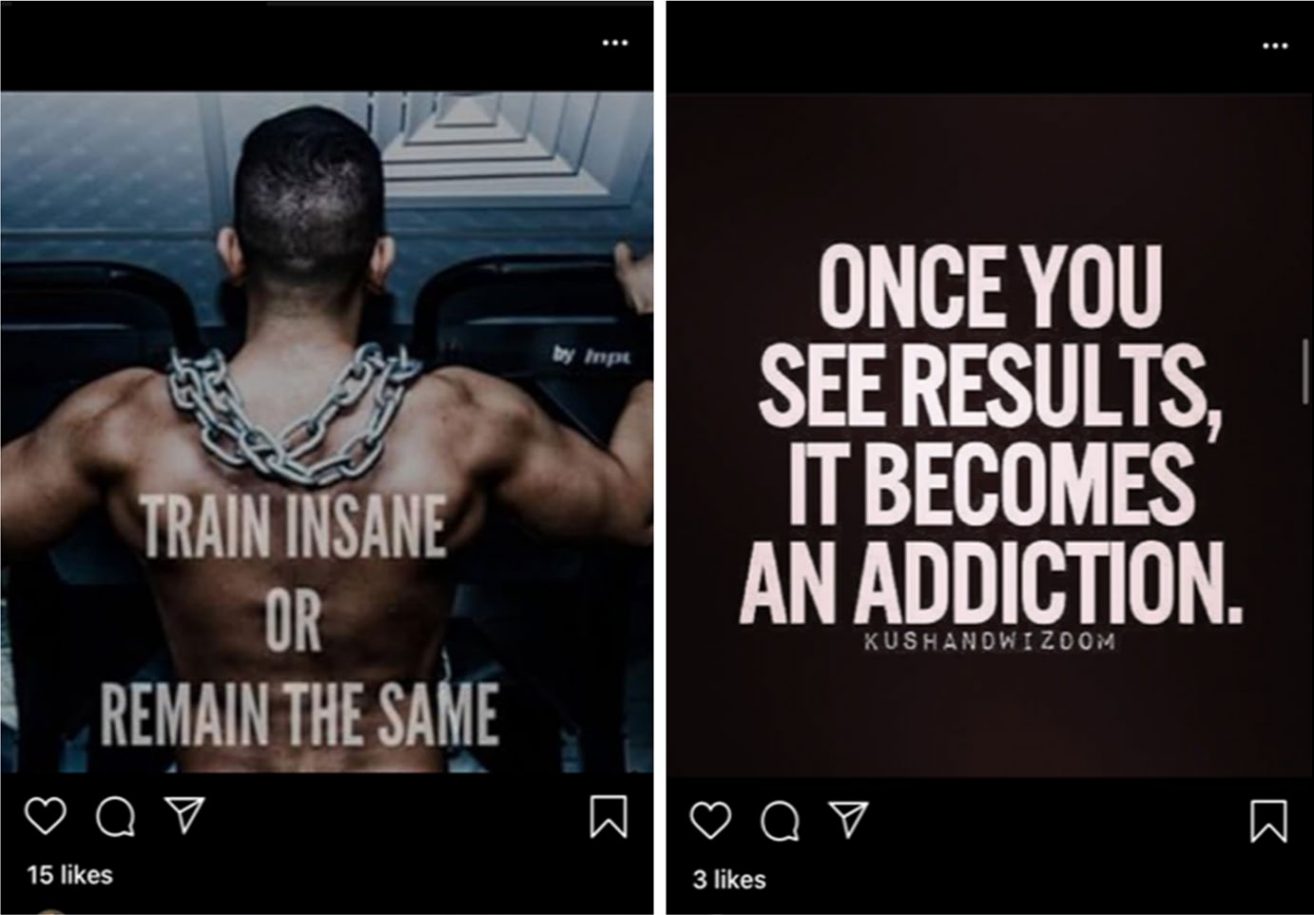
Social media reseller @Suppspharm posting content that echoes the tropes of male fitspiration accounts (08/11/19) (05/12/19)

The first tactic mobilised by social media resellers involves echoing common tropes of bodybuilding fitspiration, wherein motivational content that mirrors the vernacular and sentiment of hardcore fitness culture is posted on their social media profile, in order to appear more proximate to their customer base. An example of this behaviour is demonstrated in Fig. 3, where social media reseller @suppspharm posts two images that adhere to fitspiration’s symbolically violent message of self-improvement and bodily autonomy (Fatt et al. 2019). This post functions on several levels. Most simply, the image attempts to ‘signal’ congruence between potential customers and the seller by employing the messaging of self-improvement through bodily toil, to which members of the hardcore fitness community adhere (De Jesus et al. 2015). By mobilising this sentiment, the supplier signals their membership to the online fitness community and attempts to foster a sense of collective purpose and increased trust (Hämäläinen 2019; Bakken 2021). This reflects van de Ven and Koenraadt’s (2017: 52) finding that online sellers employ the ‘behaviours and characteristics’ of the fitness community in order to bolster trust as part of a model of responsible vending.

However, the utilisation of fitspiration-style content may also function to stimulate a sense of bodily lack and self-awareness in the prospective consumer, leaving them more susceptible to purchasing IPEDs. Whether this is @suppspharm’s intention is questionable, however the content certainly echoes the bodily ideals promoted on platforms like Instagram (Tiggemann and Zaccardo 2018) and therefore could lead to an increased desire for biomedically enhancing products.

**Fig. 4:**
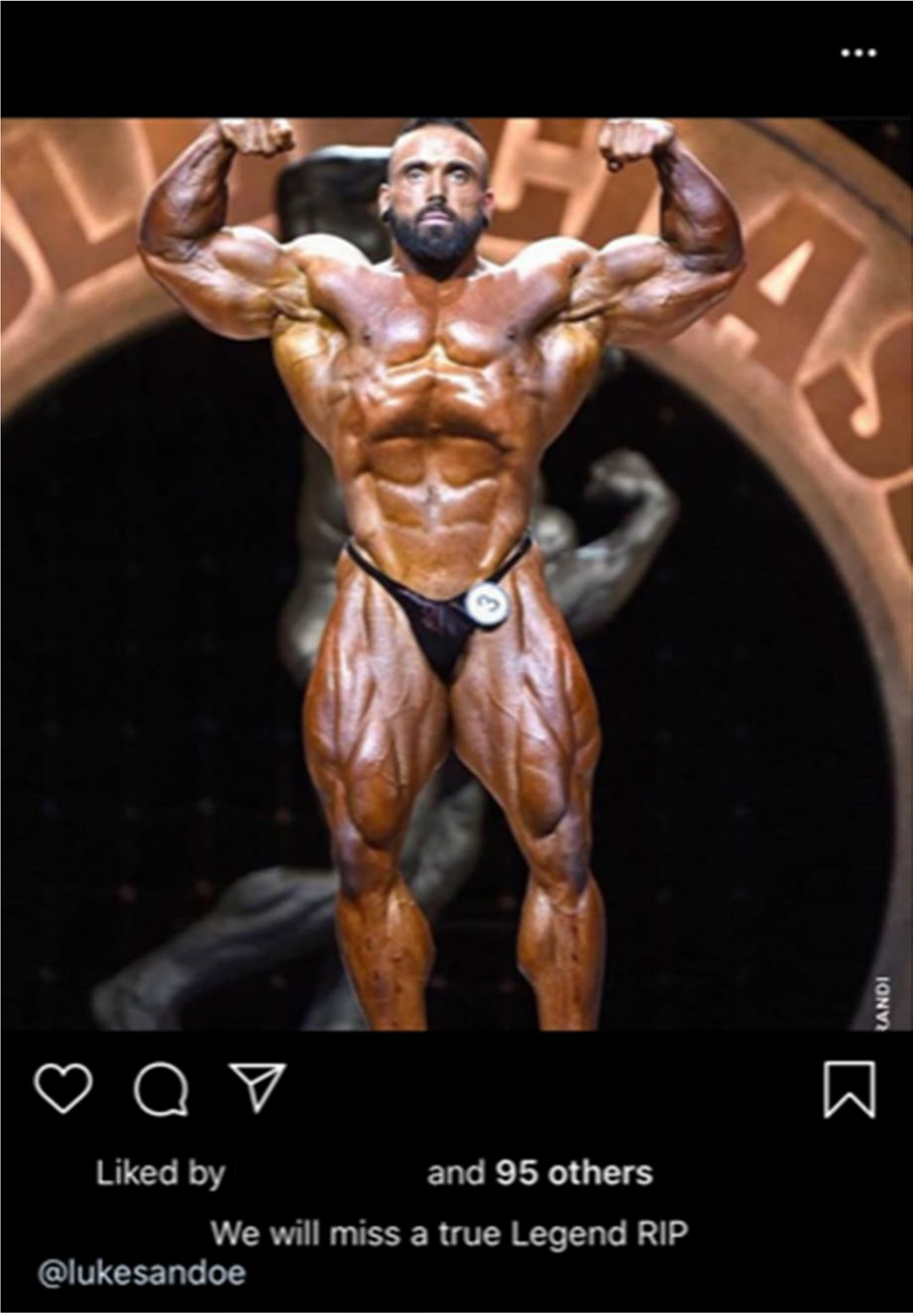
Social media dealer @superroids demonstrating their allegiance to the bodybuilding community with a post reacting to IFBB Pro Luke Sandow’s untimely death (09/05/20)

**Fig. 5:**
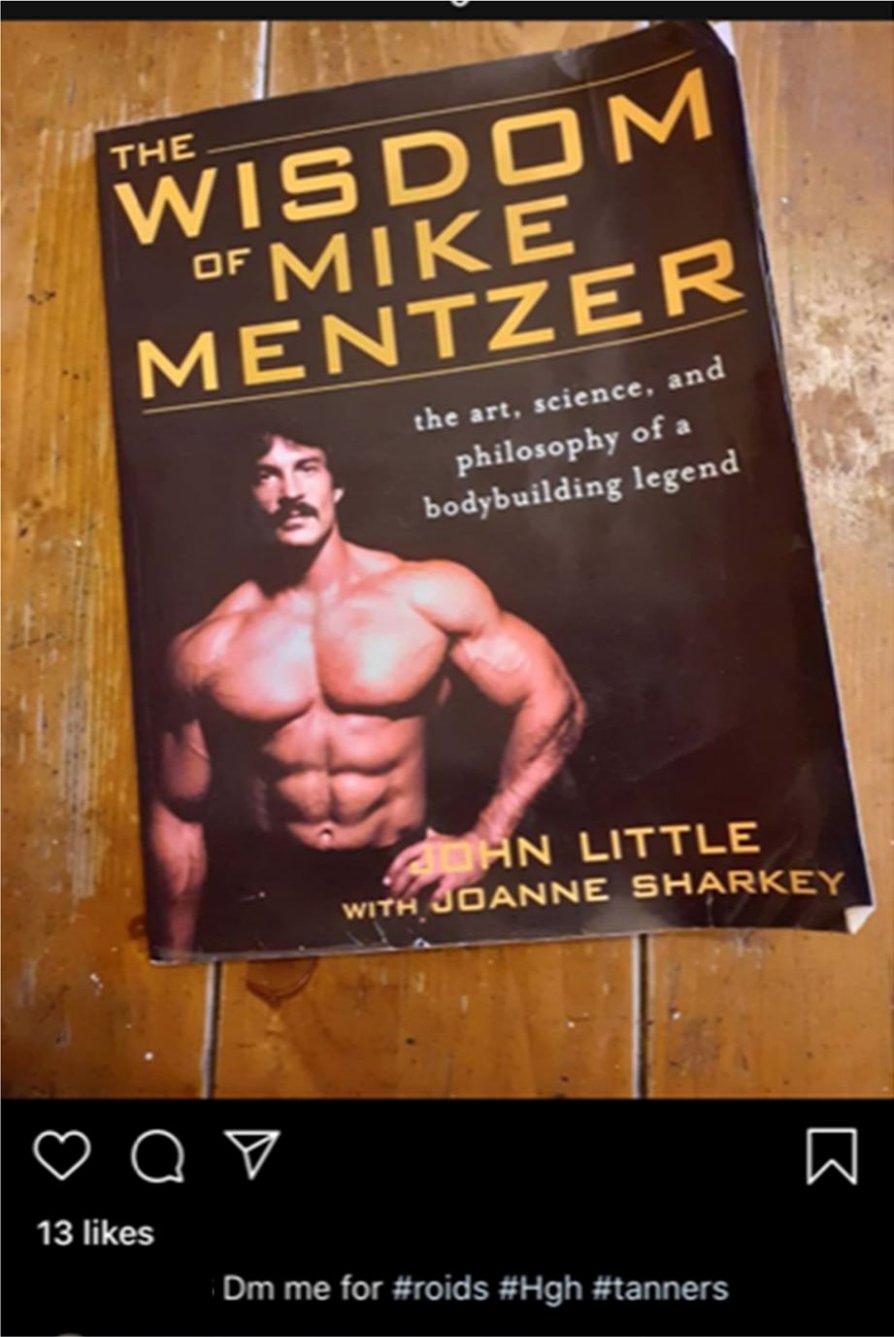
Social media seller @tren2000 signalling bodybuilding group membership through their choice of literature (05/05/20)

Further, commonality is cultivated through engagement with the sport of bodybuilding, as is demonstrated by seller @superroids’ lament to well-known IFBB Pro Luke Sandoe, who passed away in May 2020 (see Fig. 4). By positioning themselves within the online bodybuilding community, @superroids is able to mirror the interests of their customers and, similarly to UGLs’ deployment of sponsored athletes, frame their operation as somewhat akin to social supply (Moyle et al. 2013; Begley et al. 2017). This is again reflected in Fig. 5, in which @tren2000 shares an image of a well-known bodybuilding manual (Fincoeur et al. 2015; van de Ven and Koenraadt 2017).

Though this self-presentation could be construed as genuine, it seems likely that the posts are primarily a means of maximising customer engagement and, ultimately, sales. The ploy can be understood in relation to the inherent challenge of being perceived as legitimate and building trust with prospective consumers online as, given the technologically mediated interactions that the sellers rely upon, they must construct enough cultural capital in their posts to convince consumers of their trustworthiness (Koenraadt 2018). von Lampe and Ole Johansen (2004) note that kinship with customers greatly increases the initial trustworthiness of a drug supplier, and thus @superroids and @tren2000 clearly mobilise this shared connection to enhance their proximity to their customers. Ultimately, the suppliers’ active efforts to appear culturally embedded speak to the shift away from community supply models (Fincoeur et al. 2015), as such tactics would not be necessary for more traditional social-commercialist or minimally commercial dealers (Coomber and Moyle 2014).

### Self-objectification and bodily capital

**Fig. 6:**
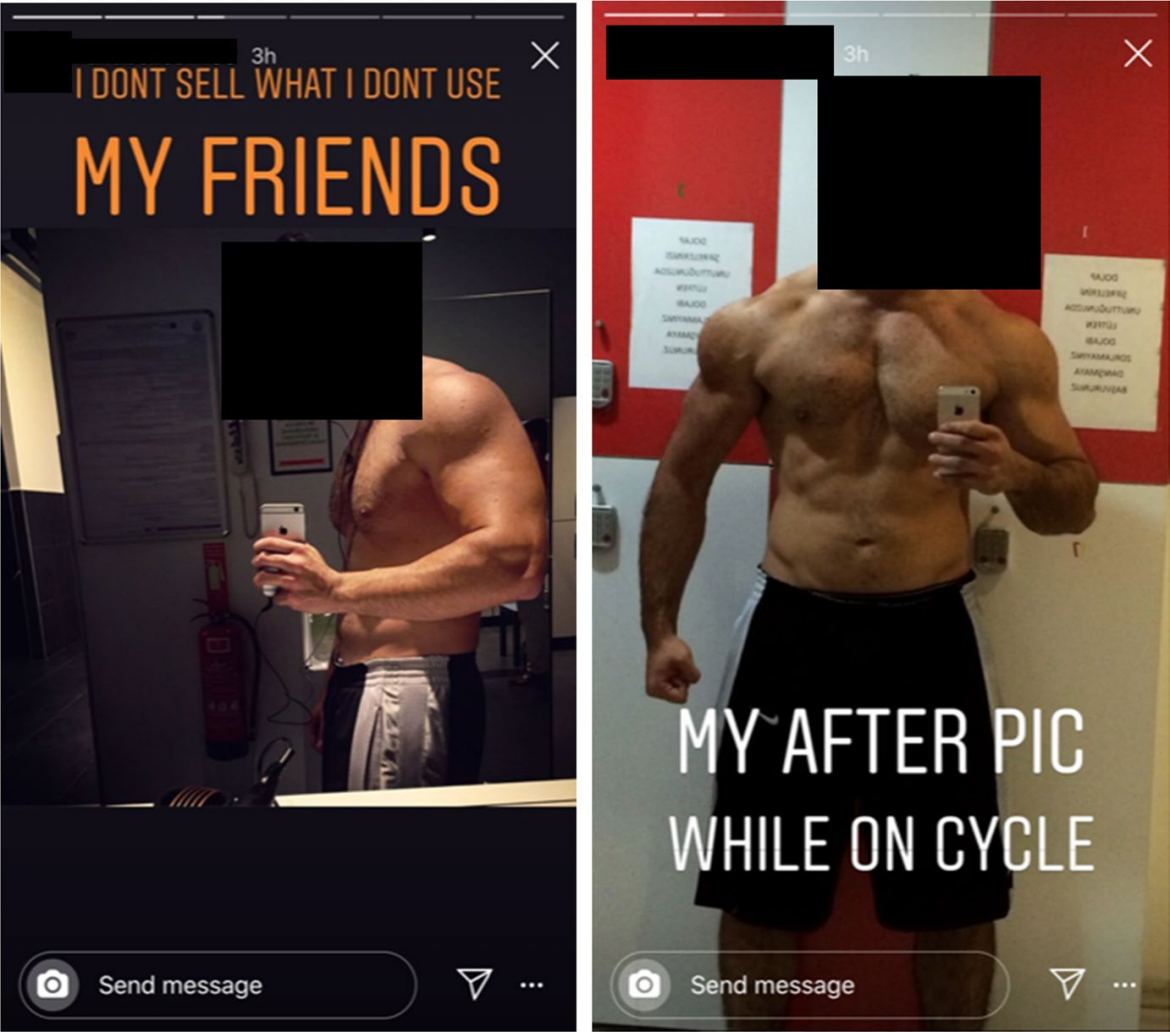
Turkish IPED seller @gear4u66 posting pictures of his physique in order to market his products (13/11/19)

Perhaps the most striking means by which IPED sellers position themselves alongside their customer base is a utilisation of their own bodily transformations. This practice is exemplified by Turkish supplier @gear4u66, whose Instagram story shows his ‘*after pic while on cycle*’, in order to simultaneously promote himself as a genuine member of the fitness community and highlight the potency of his products (see Fig. 6). Clearly, if the image is his physique, @gear4u66 is employing his ‘boosted’ bodily capital (Kotzé and Antonopoulos 2019) to increase the proximity between him and his client base, demonstrating ‘signs’ of true authenticity (Gambetta 2009; Bakken, 2021). More pertinently, the seller states, ‘*I dont sell what I don’t use my friends* [sic]’, vouching for both the safety and effectiveness of his IPEDs, leveraging his consumption within his marketing strategy in an effort to mirror his customer’s behaviour in a form of ‘digital prosumption’ (Hall and Antonopoulos 2016; Hall 2019). Linguistically, @gear4u66’s choice to address his ‘*friends*’ is telling, as it alludes to his constructed identity as a peer from whom users can not only purchase IPEDs, but also share a journey of bodily enhancement (van de Ven and Mulrooney 2017). In doing this, it appears that the reseller is aware of the inherent mistrust in online sellers and is therefore attempting to emulate the tropes of social, community-based supply (van de Ven and Koenraadt 2017).

@gear4u66’s post is also enlightening in regard to his awareness of regulation. It appears that this seller all but disregards the threat of the regulatory authorities (Gibbs et al. 2022b) and is instead wholly concerned with dispelling any perception that he may not be authentic. This contradicts van de Ven and Koenraadt’s (2017) contention that online IPED sellers tend not to partake in drug consumption or training. However, given the similarity of the licit and illicit ergogenic aids markets online, it is unsurprising that this practice has bled from PTs and other fitness professionals into the illicit IPED market.

### Transformation photos

**Fig. 7:**
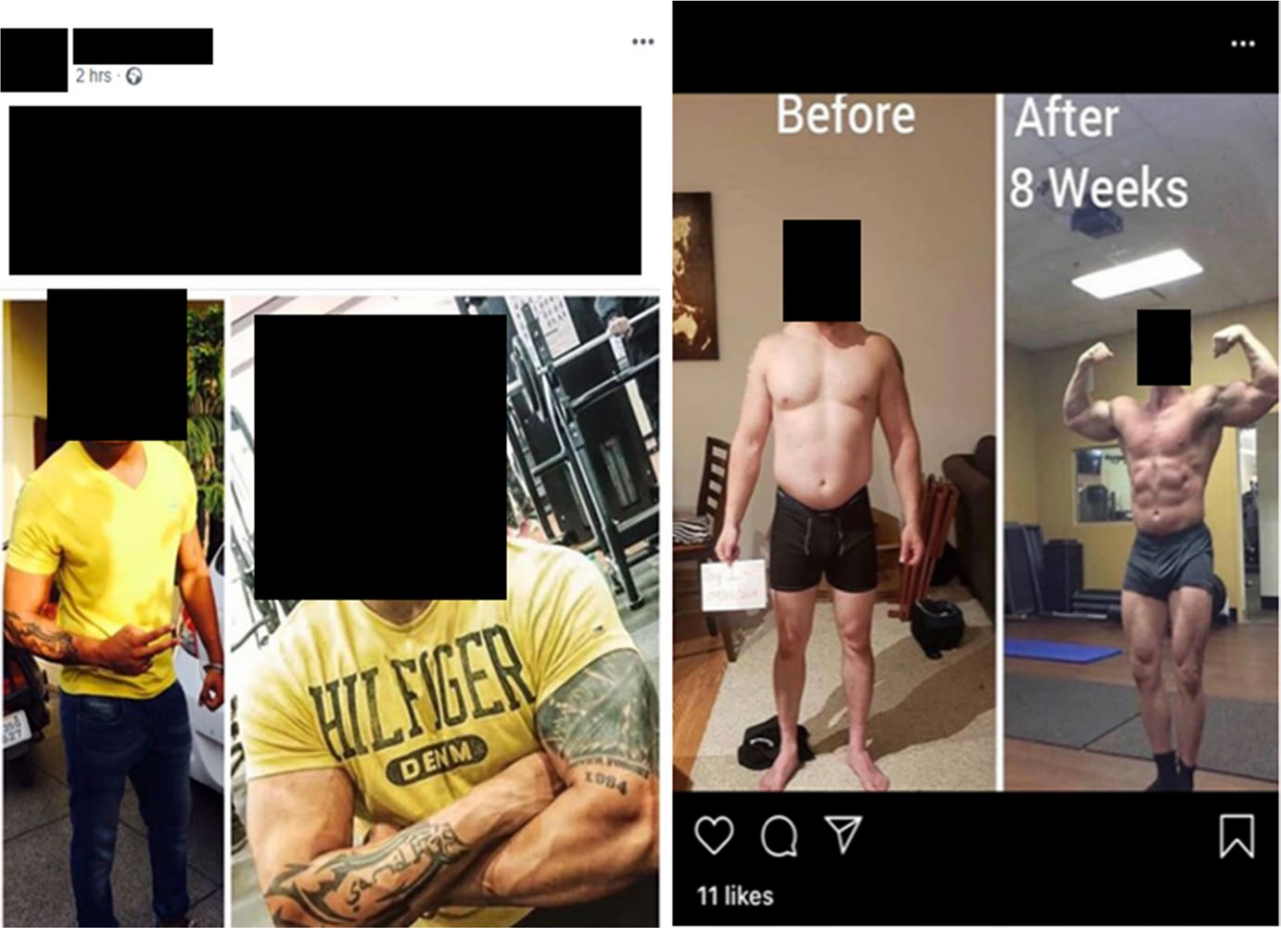
Roids Asia (left) (17/06/20), @big_dog02 (right) (07/07/20) posting transformation pictures of their clients to promote their products

**Fig. 8:**
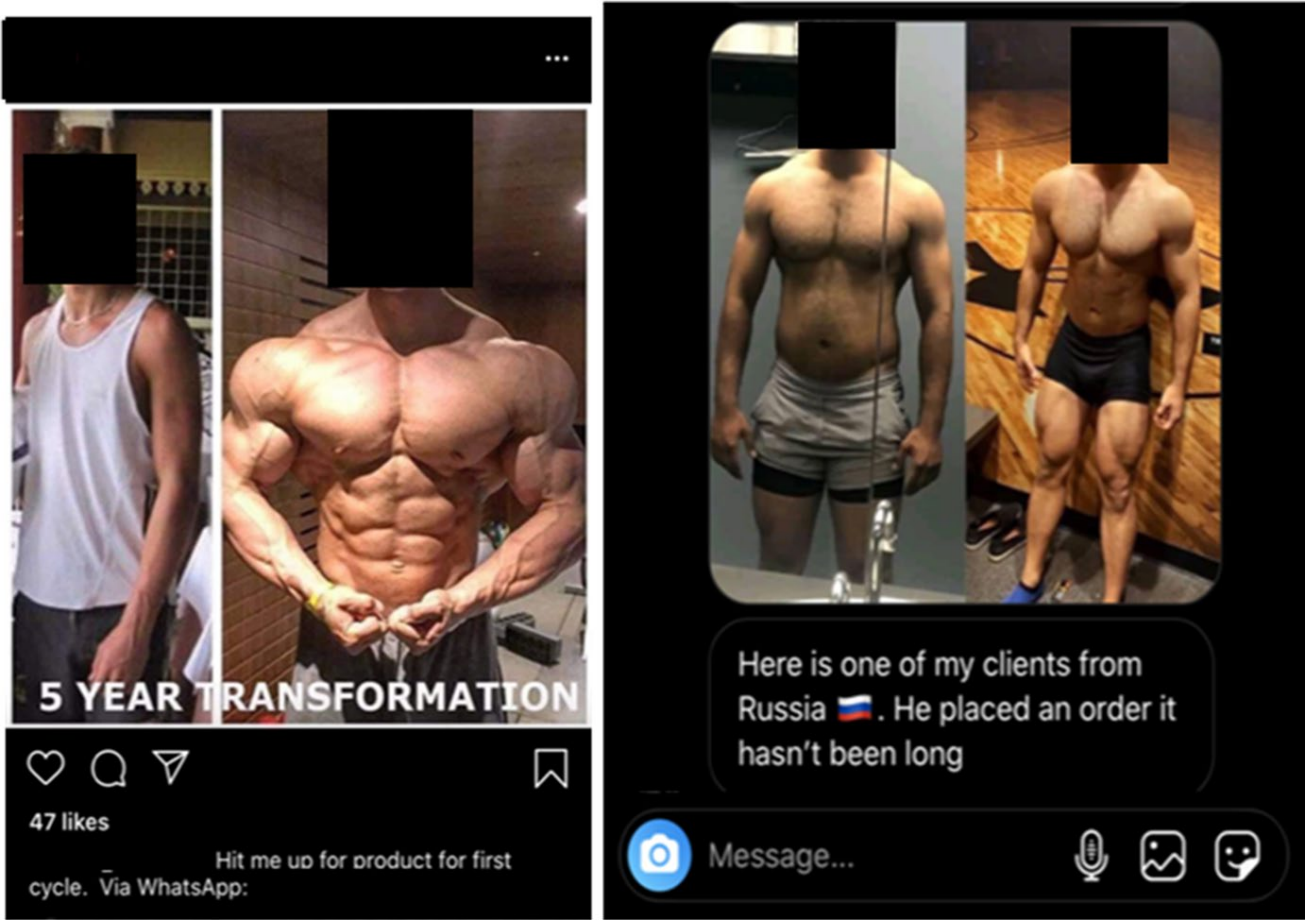
@legalisesteds (left) (22/06/20) showcasing their customer’s purported five-year transformation. @strong_supps (right) sending an image of his Russian client in a covert customer interaction (21/05/20)

An additional marketing practice used by resellers is the customer transformation, or ‘before and after’, picture (Parasecoli 2005), more commonly used in the licit fitness industry to prove the effectiveness of a product or exercise regime (Bakken and Harder 2022). This is one of the most striking similarities between the online IPED marketplace and behaviours exhibited by many personal trainers as, without the captions attributing the purported transformations to IPEDs, these could be mistaken for those of legitimate fitness professionals (Hockin-Boyers et al. 2020; Basabain et al. 2021). The below posts by Roids Asia, @big_dog02 and @legalisesteds (Fig. 7) emphasise these parallels. Users’ physiques are employed here not only to illustrate the transformative effect of the substances, but also to give the impression that the seller has established a substantial and engaged customer base. This is particularly evident in the conversation with @strong_supps (Fig. 8), where the seller shares an image of one of his ‘*clients from Russia*’, showcasing both his globalised operation and the apparent efficaciousness of his products.

However, as has been widely documented, images mediated through social media may have been doctored and filtered (Bell 2019; Leaver et al. 2020), and therefore sellers are able to manipulate their customers’ ‘after’ photos in accordance with the social media platform’s injunction of maximal aesthetic attractiveness (Schreurs and Vandenbosh 2022). More troublingly, due to the lack of regulation, the legitimacy of the images in Figs. 7 and 8 cannot be confirmed and therefore the ‘transformations’ may in fact not be the sellers’ customers at all. This, again, illustrates the inherent scope for deception on social media sites, as even trust-building tactics may draw upon fakery in order to vie for customers’ attention. Further, given the increasingly democratised, ‘open to all’ nature of the IPED online market (Antonopoulos and Hall 2016), customers that lack community-specific knowledge may not understand that both @big_dog02’s and @strong_supps post clearly show off-peak physiques compared to lean, stage-ready bodies.^4^ Though IPEDs presumably enhanced both customers’ prep, the ‘transformations’ presented look to have been brought about through fat loss rather than any real muscular hypertrophy. This speaks to the lowered barriers of entry to IPED supply brought about by online trade and how this lack of cultural capital can be exploited by sellers.

Despite this fakery, the production of transformation images allows the seller to simultaneously advertise their products and dispel the distrust within the marketplace. Though the physiques in Fig. 8 vary considerably, the ‘before’ image functions as a point of anchorage with which prospective customers can empathise. Therefore, whilst the ‘after’ photo would previously have seemed unattainable, they are presented with the means of capturing this idealised physique by the seller (much like Carl’s role for the various UGLs he was sponsored by). Finally, transformation photos like @big_dog02’s, which depicts the purported effects of an eight-week cycle, connect with both the medicalised subject’s longing for a ‘*quick fix*’ (Tim) and social media sites’ directive of ‘*instant gratification*’ (David). By explicitly quantifying the effects of the substances on sale, online sellers can tap into the ‘consumerist ethic of immediate gratification’ (Kraska et al. 2010: 181) that not only pervades the enhancement drugs market, but consumer capitalism at large. Thus, the nexus between increased consumer medicalisation and social media’s ethic of immediacy is evident, as these factors coalesce to perpetuate consumer desire and IPED sales.

### Customer feedback

**Fig. 9:**
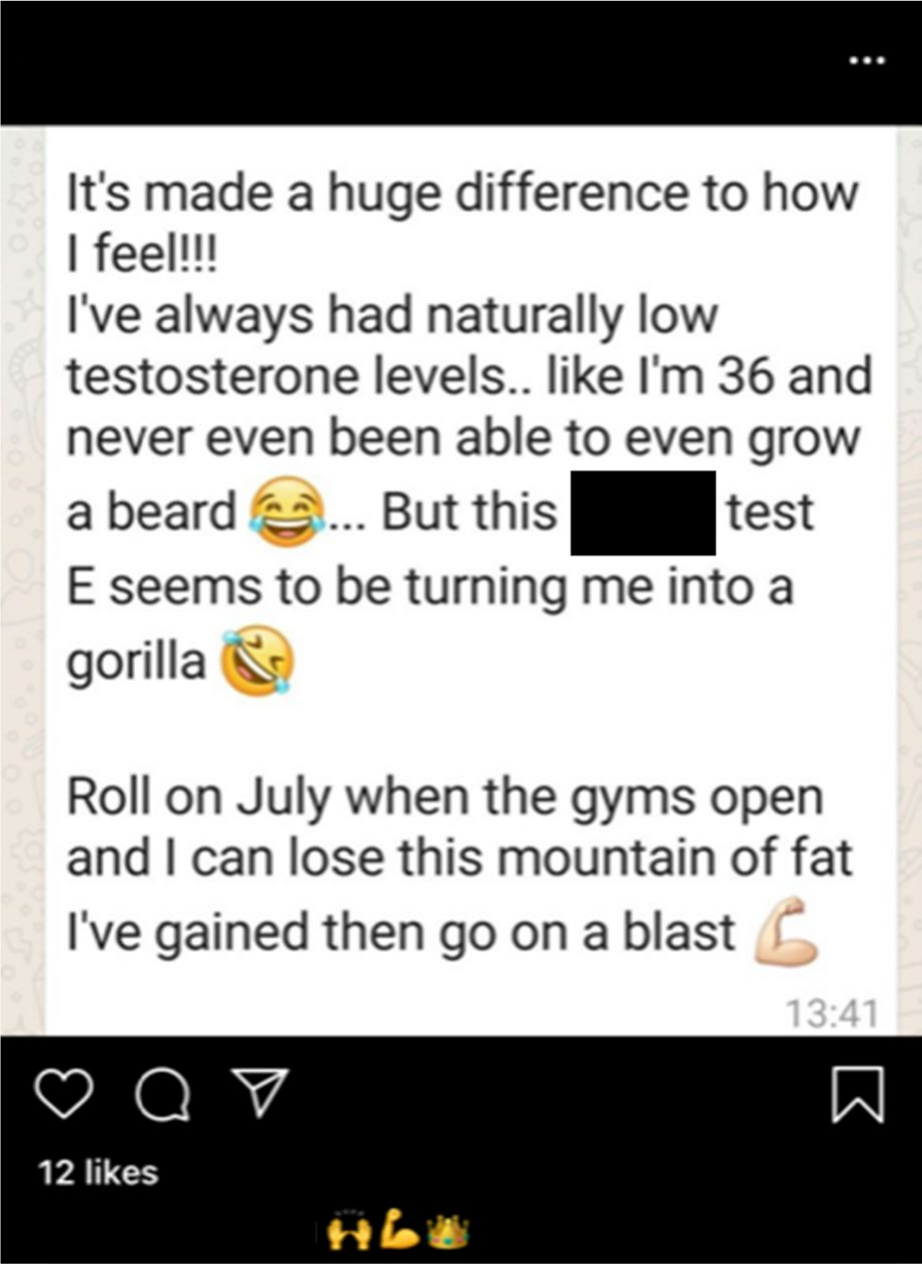
Online seller @UKanaboliclord showcasing a client’s positive review of their products via an Instagram post (23/06/20)

**Fig. 10:**
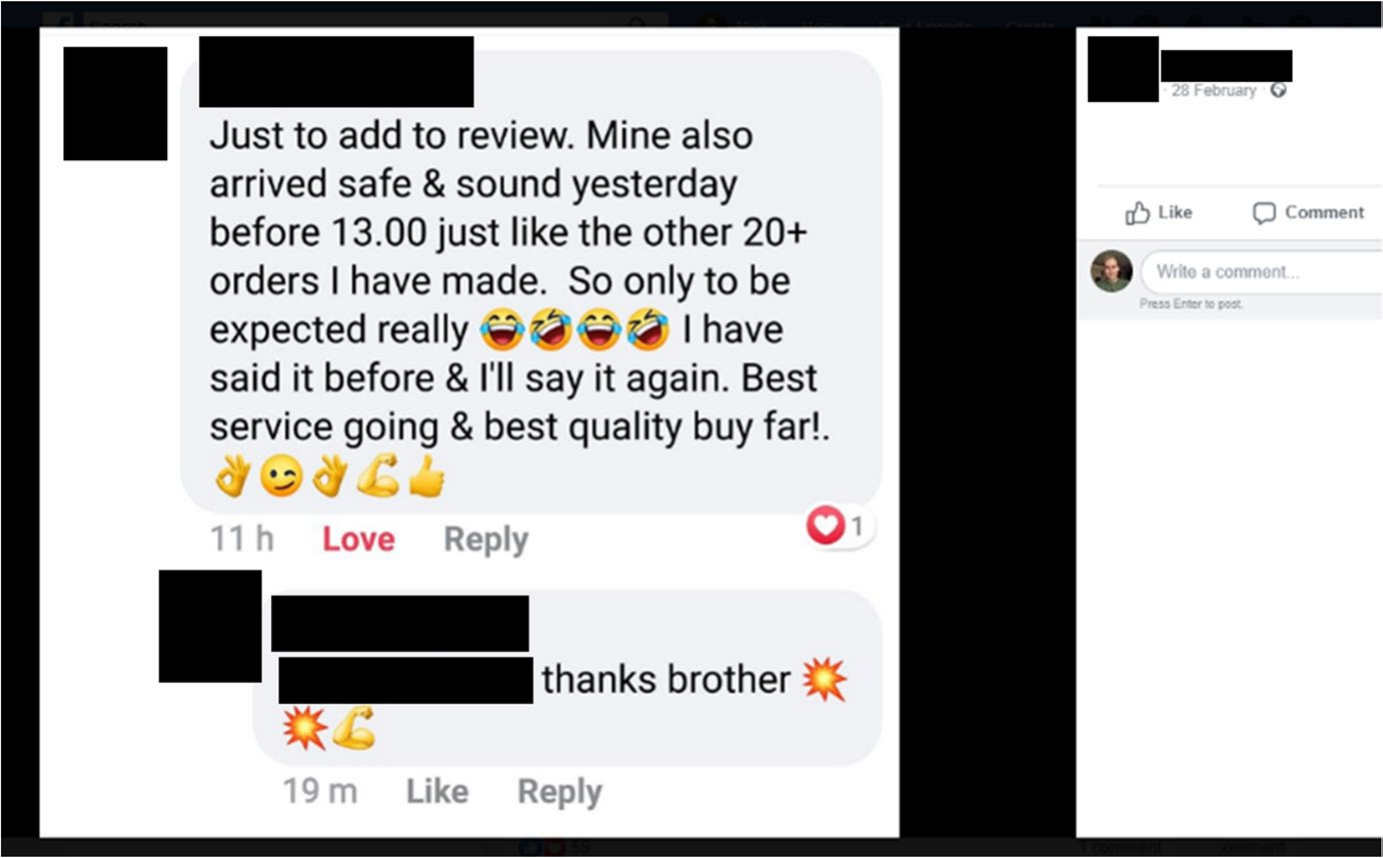
Facebook seller Bobby Apex sharing a repeat client’s review (08/04/20)

The penultimate utilisation of social media platforms by online suppliers is the sharing of customer feedback in an effort to prove the legitimacy of their operation. Just like the use of transformation pictures, this echoes legitimate practices by fitness professionals and mainstream companies, whereby eWOM marketing is utilised to complement the sellers’ efforts (see Figs. 9 and 10). This is reminiscent of Mackey and Nayyar’s (2016) contention that rogue online pharmacies draw upon customer testimonials and illustrates the crossover between the illicit medicine industry and the IPED marketplace. This is particularly evident in @UKanaboliclord’s screenshot of a customer’s positive feedback (see Fig. 9), which aims to signal the supplier’s legitimacy and authenticity to prospective customers (Koenraadt 2018, 2019). Here, the client’s review betrays the seller’s target market, as he references his ‘*naturally low testosterone*’ which has, prior to starting his cycle, inhibited the man from successfully growing a beard. This, again, speaks to a mainstreaming of the IPED market, where products are being advertised beyond the hardcore fitness population and instead targeted at laypeople seeking to improve their overall wellbeing (Kimergård 2015; Underwood et al. 2021; Dunn et al. 2021; Harvey et al. 2021; Turnock 2022).

### Sales and seasonal offers

**Fig. 11:**
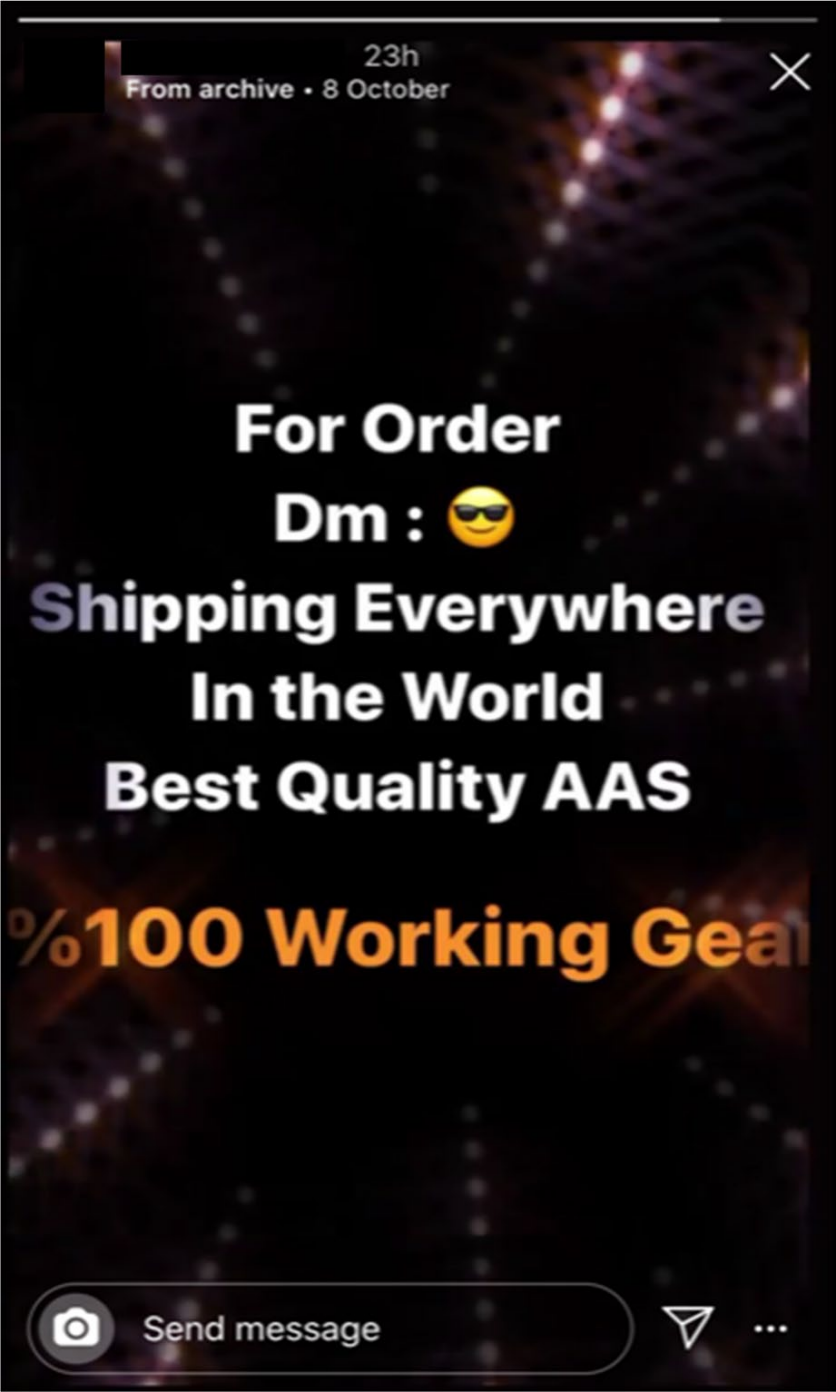
@muscledelivery using the Instagram story feature to promote their products and advise customers of the protocols of social media transactions (08/10/19)

The final social media marketing strategy employed by online IPED sellers is the advertisement of seasonal offers and special discounts, often utilising the ‘story’ feature on Facebook and Instagram. This is exemplified by Instagram supplier @muscledelivery (Fig. 11) as they draw upon the platform’s marketing affordances to produce an eye-catching advertisement for globally shipped ‘*100% Working Gear*’. The story acts as a guide to prospective customers, as the seller instructs the viewer to privately message the account with a specific emoji to order. This alludes to the lack of official protocol in the social media IPED market as, unlike online pharmacy websites, the customer may not be aware of the norms of social media market interaction given its relative infancy (Demant et al. 2019).

The use of such advertising echoes the licit marketing industry’s focus on content engagement (Ashley and Tuten 2015; Lee et al. 2018), as the story feature allows buyers and sellers to fast-track to the messaging ‘backstage’ (Goffman 1959) through the text box below the advert. The role of technology in facilitating the instantaneous mass dissemination of content is also evident here as, unlike traditional offline sellers, @muscledelivery is able to reach all of their followers with one simple marketing message, rather than soliciting individual users. This tactic echoes myriad legitimate businesses’ use of social media (Tuten and Solomon 2018) and emphasises the centrality of platforms like Instagram not only in the IPED market, but in contemporary economic development as a whole.

**Fig. 12:**
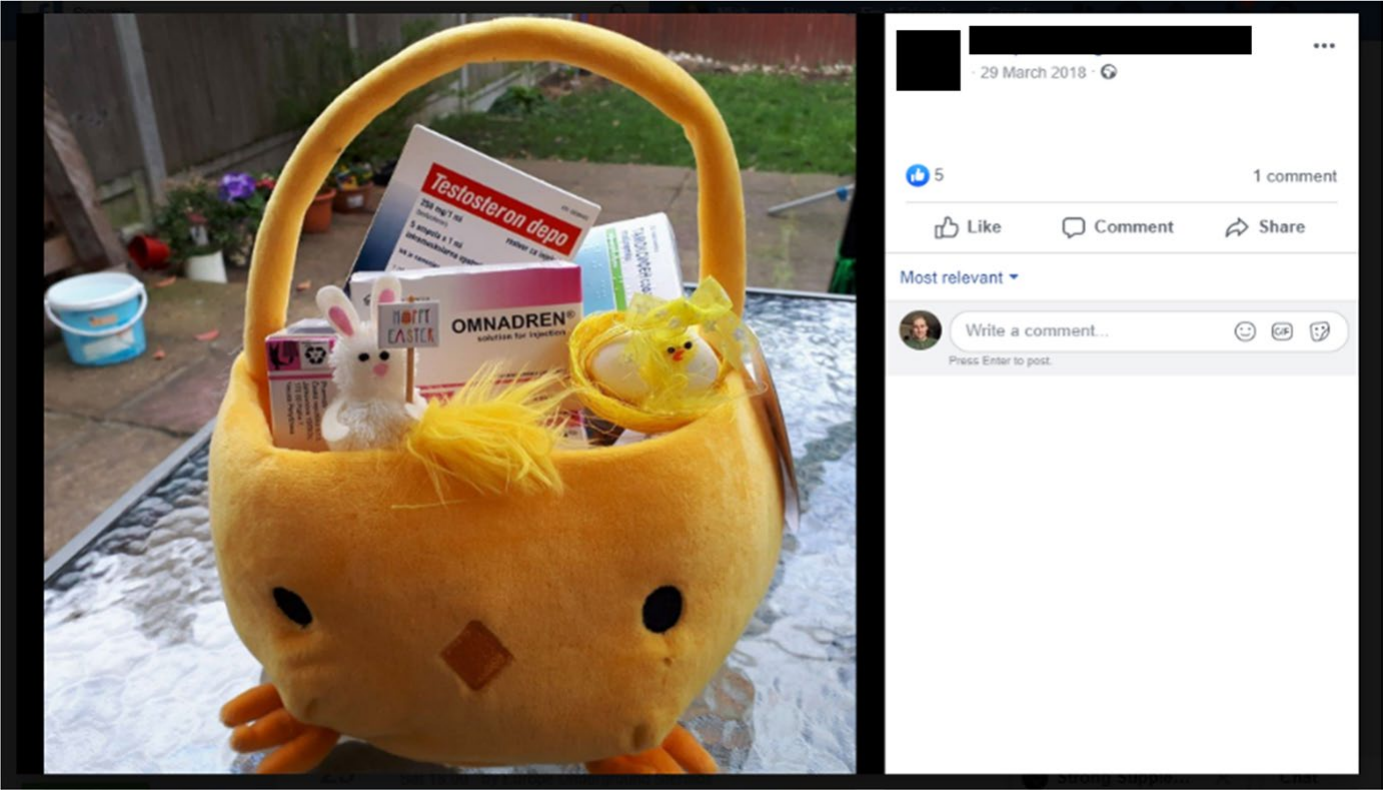
Facebook supplier Cyber Steroids mirroring the legitimate economy by offering an Easter sale (07/04/20)

**Fig. 13:**
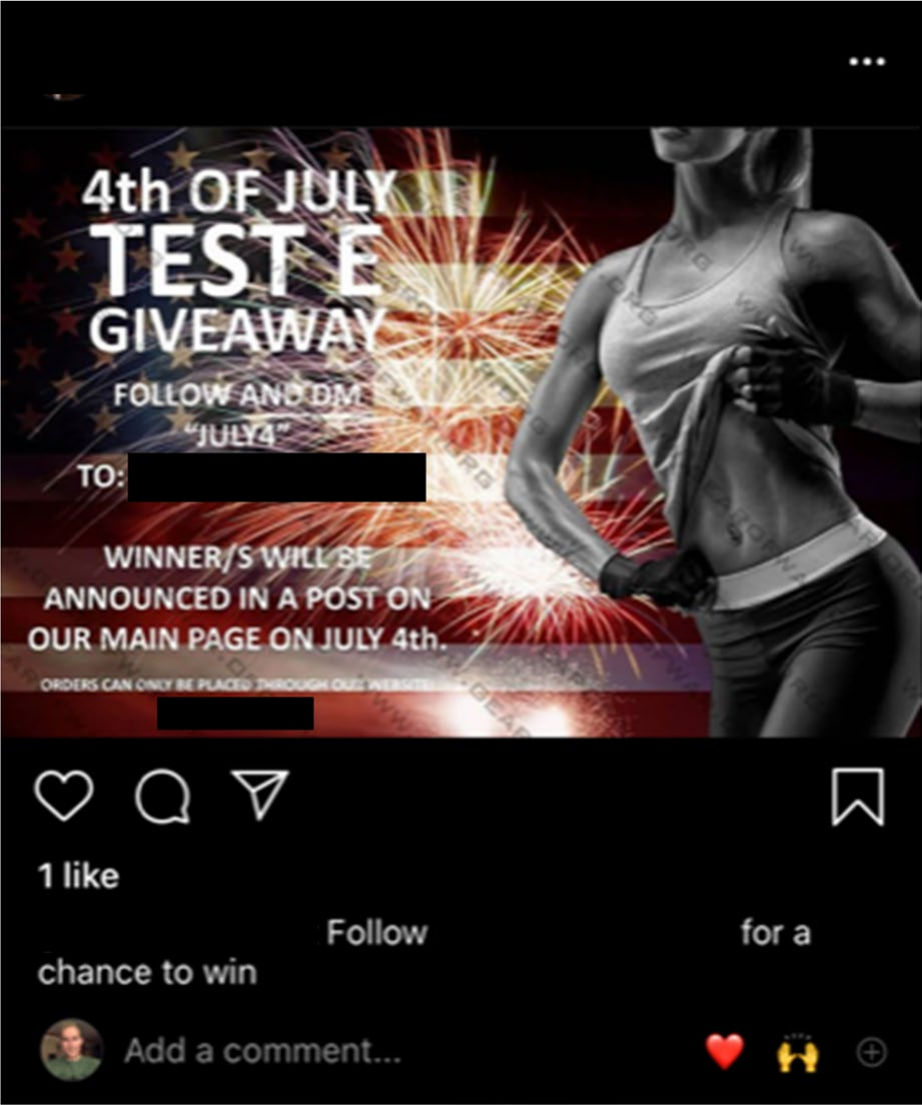
American supplier @roids_USA announcing a 4.^th^ July celebration giveaway (26/06/20)

**Fig. 14:**
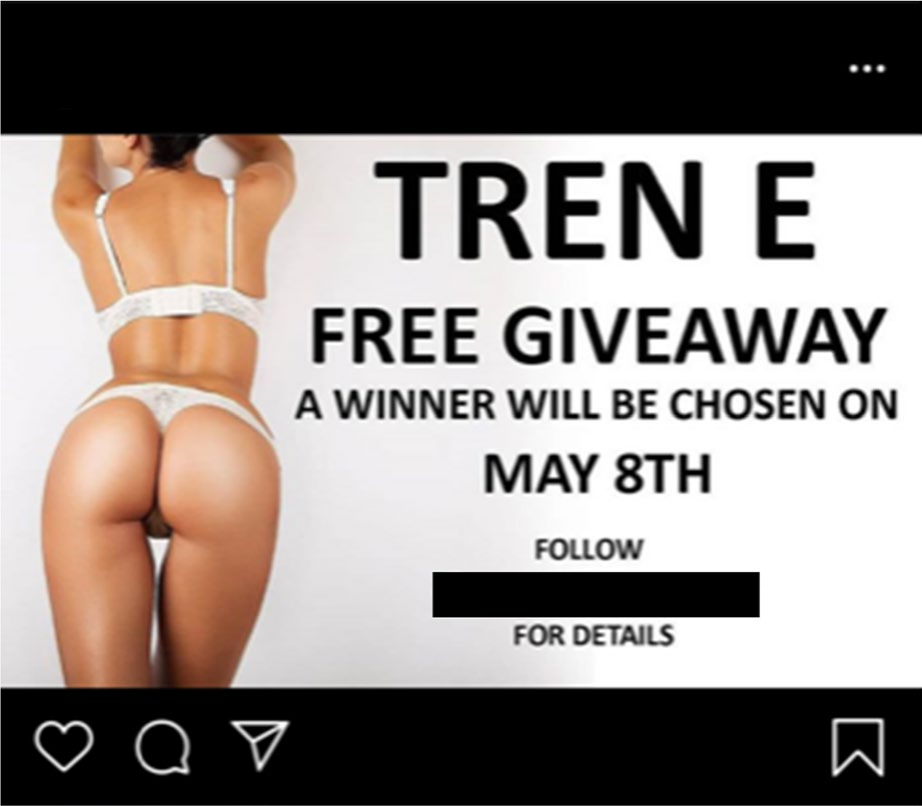
American online supplier @roids_USA presenting a free giveaway competition, using a sexualised female body to engage customers (03/05/20)

Evidence of the IPED market’s parallels with the licit economy can also be found in online sellers’ appropriation of seasonal promotions. As demonstrated in Figs. 12, 13, and 14, social media suppliers Cyber Steroids and @roids_USA capitalise upon the Easter holiday and the USA’s 4^th^ July celebrations to promote their products on social media. Whilst the nature of the promotion is not clear in Cyber Steroids’ post, @roids_USA advertises a free testosterone enanthate giveaway, provided that customers follow their page.

Finally, Fig. 12’s presentation of various AAS compounds, although amateurish in nature, clearly illustrates an attempt to curate a consumer-friendly aesthetic. Bakken and Harder (2022) discuss this in relation to licit market female cannabis influencer behaviour. They contend that a gendered showcasing of their cannabis products, including deliberate lighting and positioning, sells the lifestyle of the substances to their followers. Though not gender-specific and far from aesthetic, arguably seller Cyber Steroids deliberately employs this humorous – perhaps even kitsch – presentation to stand out in the congested market and create an element of uniqueness to their brand.

## Discussion

### Building a brand and the cultivation of trust

Consistent with the swathes of literature addressing the digital self-presentation of online illicit drug suppliers (Hämäläinen 2019; Demant et al. 2020; Bakken, 2021), the curation of a *trustworthy* brand constituted a central pillar for both types of sellers. This was variously manifested as athlete sponsorship, an emphasis on cultural proximity through bodybuilding fitspiration and self-objectification, electronic word of mouth advertising, and the display of complimentary customer feedback. The notion of building kinship with the online customer base, necessitated by the inherent distrust in ‘faceless’ online illicit drug supply (Tzanetakis et al. 2016; Bancroft et al. 2020), is important here as, echoing van de Ven and Koenraadt (2017), these strategies attempted to replicate offline peer-to-peer interactions and minimally commercial supply (Coomber and Moyle 2014). However, somewhat challenging van de Ven and Koenraadt’s (2017) overwhelmingly positive assessment of ‘responsible vending’ practices, analysis also identified a more cynical commercial underbelly to the marketing techniques. Indeed, it is not always clear whether tactics like sharing bodybuilding fitspiration and posting (supposedly) self-curated bodily content truly aim to build kinship, or instead simply intend to bolster the likelihood of sales. Though these two aims are certainly not mutually exclusive, this work draws slightly more dour conclusions about the implementation of such tactics compared to van de Ven and Koenraadt (2017).

Further, UGL representatives’ practice of employing fake accounts to present superficial ‘buzz’ (which, it should be noted, were interpreted from Rob’s anecdotal narrative rather than concrete evidence) appear downright deceptive and, ironically, undermine the very trust that many other techniques seek to nullify. This is further echoed in the diverse landscape of athlete sponsorship. On one hand, brand ambassadors like Carl, in his work for Inception, are able to offer knowledgeable best practice and other guidance (see Gibbs et al. 2022a), and yet the exploitative practices, coupled with the market’s commercially minded reaction to the pandemic, surely signal a fundamental profit motive that somewhat undermines the ‘responsible vending’ practices on offer. However, what remains, despite these questions about intent and authenticity, is a commitment to building a coherent brand that can be recognised and trusted by the consumer.

Finally, although the wider research project identified social media resellers as less inclined to reveal their identity compared to UGL representatives, the above strategies broadly challenge the awareness of the transparency paradox discussed by Tzanetakis et al. (2016) in the social media IPED market. Poignantly, it was social media resellers that were overtly posting self-objectifying content and engaging in an advanced form of ‘prosumption’ through employing their boosted physiques (Kotzé and Antonopoulos 2019). Hall (2019) navigates this development of digital prosumption expertly in relation to the wider lifestyle drug online market. She contends that the lines between producers and consumers have blurred considerably, going some way to explaining this bold marketing strategy. The reason for this openness compared to other illicit markets, it can be argued, is the relative under-policing of IPED supply and the lowly position that substances like anabolic steroids occupy in law enforcement priorities (Gibbs et al. 2022b). This is a potentially prescient finding in relation to policy and policing and adds texture to discussions about trust and branding.

### Licit/illicit market overlap

As noted, Mackenzie (2020: 2) contends that ‘[i]llegal business dances to very much the same tune as legal business’. This is certainly true of the social media IPED market, as a number of practices have been shown to have transcended the legal mainstream economy to have become embedded in the practices of UGL representatives and social media resellers. Principal amongst these is the deployment of sponsored athletes by UGLs, actors who employ calibrated amateurism (Abidin 2017) and aspects of anabolics coaching (Gibbs et al. 2022a) to furnish prospective customers with a trusted face and an authentic point of contact, with whom they can enjoy a sense of cultural proximity (Bakken and Harder 2022). More broadly, techniques like sharing ‘boosted’ transformation photos and seasonally themed sales content bely a conformity to the mainstream economy. This should not come as a surprise given that the ultimate aim of the IPED suppliers, just like any commercial actor, is to accrue capital, and social media has become a prime site through which to sell a host of licit and illicit products (Fuchs 2013). The numerous points of convergence, therefore, simply represent the most effective means of online branding and marketing for a commodity that is a low priority for law enforcement.

However, certain cultural aspects also account for the coherence of the techniques under study. As was detailed in Section "The digitised health and fitness industry"., the wider health and fitness industry has undergone a substantial digitisation in recent years (Jong and Drummond 2016) and therefore consumers of wellness products commonly ‘prosume’ on platforms like Facebook and Instagram (Hall 2019). As such, practices like social media resellers’ self-objectification and the numerous transformation photos echo tropes of users in a process of cultural reciprocity. Therefore, these social media marketing practices, it can be argued, would not have come to fruition without two symbiotic elements. First, the digital fitness culture prosumed by prospective customers, and second, the existing (highly effective) practices of licit commerce.

### Platform (mal)affordances

The final theme emanating from this exploration is the notable affordances and ‘malaffordances’ of both platforms that facilitate IPED supply. To first address the former of these, a fundamental capitalist logic undergirds the replication of mainstream marketing tactics in the social media IPED market. Facebook and Instagram, by design, lend themselves to the perpetuation of capital and the stimulation of consumer desire both as spaces of commerce (for example, in-app purchasing) and advertisement (particularly the monetisation of self-representation afforded to social media influencers (van Driel and Dumitrica 2021)). As a result, these platforms afford IPED sellers with the tools to simulate cultural proximity through the ability to post relevant content, self-objectify as an act of prosumption, mobilise sponsored athletes, and more broadly exist in the same ‘field’ (Bourdieu 1980) as their customer base. As is argued above, the digitisation of fitness is symbiotic with the burgeoning licit and illicit social media ergogenic aids market, and therefore Facebook and Instagram allow non-culturally embedded sellers (Fincoeur et al. 2015) not only access to their client base, but also a periscope into the language, self-presentation, and culture of prospective consumers. This is compounded by affordances like Instagram’s ‘story’ feature (Kurniawan 2020), which is utilised for seasonal sales and promotions as well as both platforms’ facilitation of visual content, which can be employed to share customer feedback and the host of other techniques described above.

However, whilst social media platforms have proven to be fruitful avenues for the supply of IPEDs and other illicit substances (Demant et al. 2020; Bakken, 2021), their simplicity to use and the ease with which users can misrepresent themselves is striking. True to the current literature on the inherent distrust in online illicit drugs markets, analysis uncovered multiple elements of deception by social media IPED sellers which, ironically, potentially functioned to undermine attempts to establish a trustworthy brand. UGLs’ reported utilisation of fake accounts to create ‘buzz’ certainly speak to this challenge, as well as the potentially misleading transformation photos posted by social media resellers. This ties into a broader literature on the innate fakery and manipulated self-representation on social media, with the ubiquitous employment of filters, curated lighting, posed images, and putting forward one’s ‘best self’ (Ross 2019; Tiggemann and Anderberg 2019; Kotzé et al. 2020). Therefore, whilst social media platforms may be potent marketing tools for the seller, the customer perhaps does not benefit from this development. This has potentially negative consequences for the safety of users and the regulation of the market.

## Conclusion

This work has set out to interrogate the means by which IPED suppliers utilise the social media platforms Facebook and Instagram to build a coherent brand and market their products. Set against the context of the partial digitisation of the IPED market (Turnock 2021a; Gibbs Forthcoming) and the burgeoning centrality of the online in the health and fitness industry, data from a year-long connective ethnography has been analysed to first establish a typology of social media IPED sellers, before showcasing a number of techniques employed by each. Sellers can be crudely placed into two categories: UGL representatives and social media resellers. UGL representatives, who sell on behalf of specific IPED producers, have been shown to mobilise athlete sponsorship and the creation of fake profiles and deception in order to build a strong brand and maximise their sales. On the other hand, social media resellers, who were found most prevalently on Instagram, undertook a host of marketing techniques including sharing ‘fitspiration’ content, posting self-objectifying motivational images and client transformation photos, sharing customer feedback, and utilising the platform’s affordances to advertise seasonal sales and promotions.

As has been discussed, several themes underpinned these techniques. First, consistent with existing literature (Décary-Hétu and Leppänen 2013; Holt et al. 2016; Moeller 2018; Koenraadt 2019), the primacy of building a coherent and trustworthy brand underpinned each strategy presented and, despite threats to this legitimacy posed by aspects of deception, the importance of simulating cultural proximity and customer kinship cannot be overstated. Secondly, obvious parallels and points of transcendence have been noted between the licit health and fitness industry and the social media IPED market. Particularly in practices like athlete sponsorship and the posting of transformation pictures, MacKenzie’s (2020: 2) contention that ‘[i]llegal business dances to very much the same tune as legal business’ certainly rings true. Finally, the suitability and affordances of Facebook and Instagram in facilitating the marketing of IPEDs are striking. Although the platforms somewhat stimulate the inherent distrust of social media illicit drug supply through their susceptibility to deception, the same capitalistic features cultivated by the sites to facilitate licit trade ultimately serve the illicit economy equally as well. This invites further investigation into the in-built suitability of social media platforms to illicit substance marketing, and raises concerning questions as to the regulation of illegal activity on the sites.

Ultimately, this article has addressed the lacuna in scholarship around the online marketing of IPEDs, building upon the work of Mackey and Nayyar (2016) and van de Ven and Koenraadt (2017) to present novel and prescient data that can inform scholarly understanding of illicit drugs markets, policy, and regulation. With an enhanced understanding of this landscape, and how it is facilitated and accelerated by both licit commerce and social media platform affordances, we are better equipped to understand buyer and seller motivation, and the everchanging digitised landscape of IPED supply.

## Data Availability

The data that support the findings of this study are available on request from the corresponding author, Dr Nick Gibbs. The data are not publicly available due to their containing information that could compromise the privacy of research participants.

## Footnotes

1 Prep, or ‘preparation’, is the process that follows bulking, where a bodybuilder progressively loses body fat and gains a competition-standard condition.

2 IFBB Pro refers to a bodybuilder who has been permitted into the International Federation of Bodybuilding and Fitness, meaning that they have attained ‘professional’ status in the sport.

3 In total, Carl was sponsored by three UGLs during this project’s fieldwork: Energise, Phoenix Labs and Inception.

4 Off-season describes the period of time where a bodybuilder is not competition-ready and does not have the lean condition associated with the sport. Typically, this time is spent ‘bulking’, where athletes aim to gain as much mass as possible.
